# Supplementary material for: Natural genetic variation impacts expression levels of coding, non-coding, and antisense transcripts in fission yeast
Source: Mol Syst Biol. 2014 Nov 28;10(11):764. doi: 10.15252/msb.20145123 (PMC4299605; doi:10.15252/msb.20145123)
Supplement: Supplementary file 1 — Supplementary Information [file msb0010-0764-sd1.pdf]

SUPPLEMENTARY INFORMATION

NATURAL GENETIC VARIATION IMPACTS EXPRESSION LEVELS OF CODING, NON-CODING AND ANTISENSE TRANSCRIPTS IN FISSION YEAST

Mathieu Clément-Ziza, Francesc X. Marsellach, Sandra Codlin, Manos A. Papadakis, Susanne Reinhardt, Maria Rodriguez-Lopez, Stuart Martin, Samuel Marguerat, Alexander Schmidt, Eunhye Lee, Christopher T. Workman, Jürg Bähler, and Andreas Beyer

Contents

I. RNA-seq-based genotyping 3

A. Accuracy of the genotyping 3

B. Genotyping and sequencing depth 4

II. Accounting for individual genomes improves transcript quantification 5

III. Chromosome I inversion 6

IV. Analysis of the genomic arrangement of genes linked to the swc5 locus 7

V. qPCR assessment of antisense/ sense gene expression 9

Supplementary References 11

Supplementary Tables S1 to S6 13

Supplementary Figures S1 to S22 19

I. RNA-seq-based genotyping

A. Accuracy of the genotyping

Because RNA-seq only allows the sequencing of expressed genomic regions, only half of the polymorphic sites could be directly genotyped; the genotypes of the other site were inferred. To infer the missing values, we considered the flanking polymorphisms. Flanking markers were also used to filter out potential genotyping errors. If two flanking markers indicate inheritance from the same parent it is likely that also the intermediate genomic region was inherited from the same parent. A deviation from this assumption is possible if either i) two crossovers have occurred in between the two informative flanking sites (on both sides of the polymorphic site of interest), or ii) a non-crossover is implicated (non-crossovers can lead to small haplotype blocks), or iii) complex gene conversion events took place (Mancera *et al*, 2008), or iv) it results from genotyping errors. If the flanking markers are close, such patterns are more likely to denote an erroneous genotype. This pattern with flanking markers distant of less than 50 kb, appeared 34 times when considering all the strains of the library. Interestingly, we observed occurrences at a similar frequency in the progenitor strains, for which the genotype is obviously known. This suggests that most of the 34 observations probably correspond to genotyping errors. Moreover, we estimated the probability of a double recombination in a 50 kb interval as follows. There are approximately 30-50 crossovers per meiosis in *S.pombe* and fewer non-crossovers (Egel, 2004). In addition, when observing only one segregant of a meiotic tetrad, only one crossover out of two results in a recombination. Considering the above,, we based our estimation of the probability of double-recombinations on 100 recombinations per segregant, which should be an over-estimation (even for F2). We then considered that recombinations follow a Binomial distribution with p being the probability of recombination at each base (100 divided by the genome size), and the number of trials n set to 50,000. From that we calculated that the probability of observing two or more recombinations in 50kb was 0.0078. Thus, altogether it seems plausible that alternating genotypes for marker intervals less than 50kb represent genotyping errors (see **Materials and Methods**).

The same reasoning was applied to the imputation of missing genotypes. When the flanking polymorphisms show the same segregation pattern and are close enough, we assumed that no recombination event took place between them to infer the missing genotype. This strategy may lead to few missed small recombinations, non-crossovers or complex conversion tracts. However, the comparison of the expression levels obtained when aligning the RNA-seq reads on the strain specific genome (SSG) vs reference genome (RG) suggests that the imputation of the missing genotypes is almost entirely correct. Indeed, more reads were mapped on the SSG for 98.3% of the genes that showed differences of expression quantification in this comparison. It implies that the genotyping was correct for a large fraction of this subset of genes. This

argument is even stronger since we showed many of the cases where expression appeared higher with reference genome mapping actually correspond to technical biases due to sequence homology (see below).

Note that, except rare cases (like (Bloom *et al*, 2013), genotyping of recombinant inbred populations for QTL studies has been carried out using microarrays (Atwell *et al*, 2010; Brem *et al*, 2002; Sinha *et al*, 2008) or microsatellite analyses (Williams *et al*, 2001), which led to a much lower genotyping precision than the one we achieved—here we directly sequenced almost half of the existing polymorphisms.

B. Genotyping and sequencing depth

When direct DNA sequencing is used to genotype an individual, the deeper the sequencing coverage, the more loci can directly be genotyped. However, this does not completely apply to RNA-seq based genotyping. When RNA is sequenced, variant genotypes can only be called in transcribed regions. Although most of the fission yeast genome is transcribed, only a fraction of it is sufficiently expressed to reliably call the genotype. In order to evaluate the influence of the sequencing depth on the genotyping, we have under-sampled the RNA-seq data from few samples at different scale. We then applied our complete analytic pipeline to the subsets to test the efficiency and the accuracy of the genotyping. Although the genotyping efficiency (i.e., the number of polymorphic site that can be genotyped) is affected by the sequencing depth (**Supplementary Figure S19A**), the accuracy of the genotype is good even at very low coverage **Supplementary Figure S19B**). The genotyping capacity of the proposed method reaches relatively quickly saturation at an effective depth of ~20x (here, we defined the effective sequencing depth as being the number of bases uniquely mapped scaled to the genome size). Hence, the sequencing depth achieved in our study (37.5x, on average) is sufficient for genotyping. It can be seen as limiting factor to apply the method in organisms that have a bigger genome. The sequencing depth required for accurate transcript quantification (Tarazona *et al*, 2011; Toung *et al*, 2011) may actually be sufficient for genotyping also in other species with larger genomes. Moreover, ~40% of the polymorphic sites that could not be directly genotyped correspond either to calls, which quality score felt below our threshold, or to ambiguous calls (heterozygous calls, see Methods). Such calls were not taken into account in our analysis because i) we favored accuracy over efficiency, and ii) they were not needed to successfully genotype the recombinant strains: the considerable sequencing depth of this dataset has led to saturation for almost all samples (**Supplementary Figure S19B**). There is therefore room for improving the direct genotyping efficiency by relaxing the threshold, using a genotype caller optimized for haploid genomes (note that the latest version of GATK (DePristo *et al*, 2011) implements this possibility), or by considering the genotype likelihoods of the different alleles of each variant to make dedicated genotype calls (a method close to this one using hidden Markov models has been proposed in

(Bloom *et al*, 2013) for genotyping via pooled DNA sequencing). Such developments may reduce the required sequencing depth for RNA-seq based genotyping.

The RNA-seq based genotyping method that we propose requires that the haplotype blocks contain sufficient polymorphic expressed genes, and well characterized progenitors. Whereas the recombinant cross of interest is haploid or inbred, we think that this method is usable with limited adaptation. In the case of recombinant outbred lines of diploid organisms, allele specific expression and haplotype phasing make the RNA-seq based genotyping more complicated and would require substantial additional development.

II. Accounting for individual genomes improves transcript quantification

To assess whether sequence variations affect expression quantification in recombinant libraries and eQTL detection, we compared alignments against the reference genome (RG) vs alignments against an individualized, strain-specific genome (SSG). As expected, more reads could be mapped to the SSG than to the RG. Nine percent of all transcript quantifications (all strains and all traits) were differentially quantified (at least one read difference), and 1,756 traits (27.2%) were differentially quantified in at least one sample. Only genes in which polymorphisms had been identified were expected to be affected by this bias. Indeed most genes containing a polymorphism (92.6%) were differentially quantified in at least one sample (**Figure 4a**). Consistent with our expectations, most of the affected measurements (98.3%) were increased for the SSG compared to the RG mapping (**Figure 4b**). A deeper analysis confirmed that mapping differences were mostly caused by reads that could not be mapped to the RG (**Supplementary Figure S8A**).

In a few measurements (1.7%), we observed reduced expression levels for the SSG alignments. These results were not expected, but could be explained by two reasons. First, they could correspond to genotyping errors or uncertainties. If a polymorphism was erroneously called as inherited from Y0036, mapping on the RG—which corresponds to 968 genome—would result in a greater quantification (therefore lower on SSG). The second explanation is more subtle: during the alignment we required that reads be unambiguously mapped to one region. I.e. all reads mapping to more than one region were excluded from the quantification. In some of the cases with lower expression levels, we observed when mapping to the SSG that the respective locus in the SSG was more homologous to another genomic region than the same locus in the RG. Hence, reads mapping to the respective locus in the SSG were more likely to also map to the second region and therefore got excluded from the transcript quantification (**Supplementary Figure S8B-C**).

The magnitude of quantification differences depended on the nature of polymorphisms (indels causing a stronger bias than SNPs, **Supplementary Figure S8D**) and on the density of polymorphisms (Spearman's rank

correlation ρ=0.9, $p<10^{-15}$, **Supplementary Figure S8E**). Nevertheless, the quantification differences between RG and SSG mapping exceeded 10% for only 0.49% of the measurements (all strains and all traits considered).

Next, we evaluated the impact of this distortion on *cis*-eQTL detection. We compared all *cis*-linkages of the 1,756 genes for which a difference in quantification was observed. *Cis*-linkages were more significant for RG than for SSG mapping (p-value $<10^{-9}$, Wilcoxon's signed-rank test). Moreover, when mapping to the RG, we detected 12.8% more *cis*-eQTLs than when mapping to the SSG (53 versus 47; FDR <5%). Only few genes were affected in our study, because of the small genetic divergence between the progenitor strains (**Discussion**).

III. Chromosome I inversion

A pericentric inversion of a 2.2Mb region on Chromosome I (Brown *et al*, 2011) was shared by the progenitor strain 968 but not by the other parental strain Y0036. Despite this inversion, successful meiosis and recombination took place between those two lines. We however observed an important reduction of the crossover frequency in this region. Indeed, in a majority of the segregants (32 strains, 73), no recombination happened in this region (**Figure 2**, **Supplementary Figure S6**).

Meiotic segregation and recombination in heterozygous carriers of pericentric inversions have been reported and studied for decades (Martin, 1991). They can lead to the formation of unbalanced (duplications or deficiencies) or balanced chromosomes (reviewed in (Anton *et al*, 2005; Morel *et al*, 2007). For instance in human, on the one hand several cases of cri du chat syndrome originating for chromosome 5 pericentric inversions have been reported (Dobbs *et al*, 1988; Levy *et al*, 2002), non exhaustive list), on the other hand pericentric inversion, notably of Chromosome 9, are found with a frequency of 1-2%, and are considered as polymorphisms (Anton *et al*, 2005; de la Chapelle *et al*, 1974; Kaiser, 1984). Sperm studies in heterozygote inversion carriers reported a reduction of the rate of meiotic recombination (Morel *et al*, 2007), as we observed in this recombinant library.

In cases of heterozygous pericentric inversion, meiotic crossovers require the formation of an inversion loop (twisting and folding of the inverted segment) during the synapsis of the homologous chromosomes (meiosis pachytene stage) (Anton *et al*, 2005). For balanced chromosomes to arise none or an even number of recombinations have to take place in the loop; otherwise, it leads to duplication or deficiencies (**Supplementary Figure S7**). Interestingly, only even numbers of recombinations were observed within the inverted region (**Figure 2**), and we did not find any unbalanced Chromosome I in the library. This suggests that the other recombination schemes, leading to unbalanced chromosomes were non-viable spores. We

noticed that the markers corresponding to the extremities of the inversion showed identical inheritance profiles in the segregants. As can be seen from **Supplementary Figure S8D** the balanced chromosomes can only be obtained if the 3’ and 5’ distal parts of the inverted region always originated from the same chromatid—and are therefore inherited from the same parents.

The linkage results showed that 937 eQTLs (40%) and 336 aseQTLs (16%) were falling in the inversion. However, considering the above mentioned recombination abnormalities, one could question the validity of those QTLs. Because of the lack of recombination, the genetic resolution of the inversion is poor. Moreover, there are very little inheritance differences between the markers (more than half of the segregants have no recombination inside the inversion region). Thus, linkages to a marker or to another within the inversion might be equivalent. The extreme case of this phenomenon are the most distal markers of the inversion, which are identical in terms of inheritance patterns (see above). Each was linked to more than 160 sense traits, which overlapped logically almost entirely (96%); however, they were not counted as independent linkages, but as single QTL which exact location (at either locus) could not be resolved. Hence, one could consider the entire 2.2Mb inversion region as a single locus.

Some of those QTLs could also be due to gene disruption or perturbation at the border of the inversion. The expression of few genes located in the vicinity of the inversion break points could be severely modified directly (disruption of the sequence of genes, promoters, etc) or indirectly (modified chromatin state). This hypothesis is supported by the fact that the inversion extremities are eQTL and aseQTL hotspots. Another hypothesis is that numerous polymorphisms are co-inherited because of the lack of recombination within the inversion. The tremendous effects observed on the regulation of expression could therefore be due to several molecular variations. Each one could act on few or multiple traits. Many QTLs would then be identified because of the coinheritance of the variations making the magnitude of the effects apparently greater. However, the population structure dependent QTL mapping that we have carried out should take most of these effect into account.

IV. Analysis of the genomic arrangement of genes linked to the *swc5* locus

Antisense transcripts originate from read-through transcription, overlapping transcript, bidirectional transcription, or autonomous transcription (Bitton *et al*, 2011; Chen *et al*, 2012; Ni *et al*, 2010; Pelechano & Steinmetz, 2013). The arrangement of genes with antisense transcription can be used to narrow down the potential cause (**Figure 8**). For example, bidirectional transcription would result in antisense transcription for genes arranged in tandem. Antisense from overlapping genes would lead to an enrichment of convergent and divergent overlapping gene pairs. Transcriptional read-through would lead to the enrichment of convergent gene pairs. The magnitude of this effect may depend on whether the 5’-end of the read-through

gene overlaps partly with the gene on the opposite strand. However, in *S. pombe* the distinction between overlapping and non-overlapping convergent genes is dubious. Transcription termination (and polyadenylation) sites are highly variable in fission yeast (Schlackow *et al*, 2013; Mata, 2013; Gullerova & Proudfoot, 2008; Gullerova *et al*, 2011). Our analysis of gene pairs with antisense transcription revealed an enrichment of overlapping and non-overlapping convergent genes and surprisingly a depletion of genes in tandem (overlapping and non-overlapping) (**Figure 8**). Although the enrichment of convergent gene pairs is consistent with transcriptional read-through, the depletion of genes in tandem required additional analysis.

We visually observed the organization of the fission yeast genome using a browser and noticed that coding protein genes seemed to be organized in stretches of tandem pairs that alternated with stretches of convergent/divergent pairs (**Supplementary Figure 17A**), which is an indication of a non-random organization of genes in the genome together (Dávila López *et al*, 2010; Hurst *et al*, 2004; Michalak, 2008; Li & Du, 2012) . We hypothesized that it was the cause of the observed depletion of tandem genes. Indeed, if a set of genes is enriched for convergent gene pairs, divergent and tandem genes pairs should be depleted. Here, tandem pairs were fewer, but convergent pairs remained stable (**Figure 8**). In the case of convergent/divergent stretches, divergent pairs adjacent to selected convergent pairs would also be selected. To formalize this observation, we looked at repartition of “direction swapping” event in the genome, i.e. occurrences of three successive genes being in alternate directions (**Supplementary Figure 17A**). We compared the number of successive direction swaps in the fission yeast genome to an empirical null distribution obtained by permuting one million times the gene orientations. There was an excess of direction swaps ($p < 10^{-6}$, **Supplementary Figure 17B**), proving that there were more stretches of convergent/divergent pairs than expected by chance in the fission yeast genome.

Next we showed that this non-random gene organization was explaining the depletion of tandem gene pairs. We randomly selected as many convergent pairs as found among the *swc5* aseQTL target genes (853 convergent pairs, regardless of the overlaps). Then as many random aseQTL targets were picked among the chosen convergent gene pairs as there were among the real convergent pairs. We then categorized the other gene pairs thus selected (either divergent or convergent) and repeated this process 1,000,000 times.

We then compared the resulting distributions of the number of divergent and tandem pairs to the one obtained by performing the same analysis but in randomizing the genome by permuting the gene orientations. More tandem pairs and less divergent pairs were selected when randomizing the genome **Supplementary Figure 17C**). Altogether, these results show that the depletion of tandem gene pairs among the *swc5* aseQTL targets was an artifact due to the non-random organization of the *S. pombe* genome.

V. qPCR assessment of antisense/ sense gene expression

Four target genes of the hotspot 11 were chosen to perform additional exploration of their expression regulation via semi-quantitative real time PCR. We chose target genes whose sense and antisense levels were linked to the hotspot 11, and that were arranged convergently, regardless of their potential overlaps. The gene pairs were: *cdb4 / thi4*; *SPAC29E6.10c/SPAC29E6.09*; *its3 /tpp1*; *paf1/rps1602* (the first indicated gene of each pair was the *swc5* target gene). Only the pair *cdb4/thi4* presented substantial overlap between them, with the annotated 3'-UTR of *thi4* covering the entire coding part of *cdb4*.

The experiments were design to interrogate the sense and antisense levels of the studied genes (which sense and antisense expression levels were linked to of *swc5*), the sense levels of the other convergent genes, and to detect potential read-through level (**Methods**, **Supplementary Figure 16A**). As expect for *cdb4*, none of the antisense levels were measured in a part of the gene that overlaps (according to annotations) with the other convergent gene. All experiments were performed in biological triplicates. In addition, particular care was taken to estimate and limit the synthesis of primer-independent cDNA, which has been shown to bias the measurement of antisense levels (Haddad et al, 2007; Feng et al, 2012). Because we were expecting widespread changes in expression levels, we restrained our analysis to expression ratios to avoid issues due to global changes affecting the reference gene (**Material and Methods**).

Results (**Figure 7**) confirmed the QTLs: we observed an increase of antisense to sense ratio in a pool of segregants carrying wild-type *swc5* compared to a pool of segregants carrying *swc5-fs*. These differences also existed among the parental strains for all but one gene (*cdb4*), a case of transgressive segregation, which implies that other genetic factor play a role, as detected in the aseQTL analysis (in addition to the swc5 linkage, the antisense level of *cdb4* was linked to another locus).

Moreover, the results in the other studied deletion strains are in agreement with the hypothesis that the effect of the hotspot 11 goes through H2A.Z. We observed an increase of the antisense to sense ratio in all cases, except for two genes in the *Δswc5* strain. In these two cases, the inconsistency may be due to either other genetic variants in *cis* of *swc5* or to the effects of auxotrophic markers in the *Δswc5* strain (auxotrophic markers can potentially widely affect gene expression (Brem *et al*, 2002)).

In the case of the gene pair *cdb4/thi4*, the increase of the antisense to sense ratio observed in *cdb4* could be simply due to an increase of the expression of *thi4* (the gene convergent to *cdb4* on the other strand, gene B in **Supplementary Figure 16A**), since these genes are overlapping (the 5'UTR of the *thi4* overlap the CDS of *cdb4*). To assess this possibility we looked that the ratio of the antisense levels of *cdb4* (measured in the CDS of *cdb4*) on the sense level of *thi4* (in the CDS of *thi4*). Results indeed show an increase of this ratio following

the antisense to sense ratio thus demonstrating that other mechanisms than gene overlaps are involved (**Supplementary Figure 16B**).

Finally, to assess and quantify read-through transcripts, we performed qPCR in the gene body of the gene opposite to the *swc5* QTL target (gene B, **Supplementary Figure 16A**) using the RT product of the antisense of the *swc5-fs* target genes (gene A, RT using AS-A primer, in **Supplementary Figure 16A**). These experiments were not conclusive even in conditions for which read-through transcription had been shown to take place (Δ*pht1* and Δ*pht1*Δ*clr4* strains). The levels obtained were too close to the background to allow for drawing any conclusions. This neither confirms, nor invalidates that read-through transcription takes place. Besides the special experimental procedure we applied to circumvent the known issue of qPCR in antisense expression measurement (Haddad *et al*, 2007; Feng *et al*, 2012), we reached the limit of the method.

Supplementary References

Anton E, Blanco J, Egozcue J & Vidal F (2005) Sperm studies in heterozygote inversion carriers: a review. *Cytogenet. Genome Res.* **111:** 297–304

Atwell S, Huang YS, Vilhjálmsson BJ, Willems G, Horton M, Li Y, Meng D, Platt A, Tarone AM, Hu TT, Jiang R, Muliyati NW, Zhang X, Amer MA, Baxter I, Brachi B, Chory J, Dean C, Debieu M, de Meaux J, et al (2010) Genome-wide association study of 107 phenotypes in Arabidopsis thaliana inbred lines. *Nature* **465:** 627–631

Awadalla P (2003) The evolutionary genomics of pathogen recombination. *Nat. Rev. Genet.* **4:** 50–60

Bitton DA, Grallert A, Scutt PJ, Yates T, Li Y, Bradford JR, Hey Y, Pepper SD, Hagan IM & Miller CJ (2011) Programmed fluctuations in sense/antisense transcript ratios drive sexual differentiation in S. pombe. *Mol. Syst. Biol.* **7:** Available at: http://www.nature.com/msb/journal/v7/n1/full/msb201190.html [Accessed January 24, 2013]

Bloom JS, Ehrenreich IM, Loo WT, Lite T-LV & Kruglyak L (2013) Finding the sources of missing heritability in a yeast cross. *Nature* **494:** 234–237

Brem RB, Yvert G, Clinton R & Kruglyak L (2002) Genetic dissection of transcriptional regulation in budding yeast. *Science* **296:** 752–755

Brown WRA, Liti G, Rosa C, James S, Roberts I, Robert V, Jolly N, Tang W, Baumann P, Green C, Schlegel K, Young J, Hirchaud F, Leek S, Thomas G, Blomberg A & Warringer J (2011) A Geographically Diverse Collection of Schizosaccharomyces pombe Isolates Shows Limited Phenotypic Variation but Extensive Karyotypic Diversity. *G3 GenesGenomesGenetics* **1:** 615–626

De la Chapelle A, Schröder J, Stenstrand K, Fellman J, Herva R, Saarni M, Anttolainen I, Tallila I, Tervilä L, Husa L, Tallqvist G, Robson EB, Cook PJ & Sanger R (1974) Pericentric inversions of human chromosomes 9 and 10. *Am. J. Hum. Genet.* **26:** 746–766

Chen H-M, Rosebrock AP, Khan SR, Futcher B & Leatherwood JK (2012) Repression of Meiotic Genes by Antisense Transcription and by Fkh2 Transcription Factor in Schizosaccharomyces pombe. *PLoS ONE* **7:** e29917

Dávila López M, Martínez Guerra JJ & Samuelsson T (2010) Analysis of Gene Order Conservation in Eukaryotes Identifies Transcriptionally and Functionally Linked Genes. *PLoS ONE* **5:** e10654

DePristo MA, Banks E, Poplin R, Garimella KV, Maguire JR, Hartl C, Philippakis AA, Angel G del, Rivas MA, Hanna M, McKenna A, Fennell TJ, Kernytsky AM, Sivachenko AY, Cibulskis K, Gabriel SB, Altshuler D & Daly MJ (2011) A framework for variation discovery and genotyping using next-generation DNA sequencing data. *Nat. Genet.* **43:** 491–498

Dobbs M, Overhauser J & Wasmuth JJ (1988) Molecular analysis of a case of meiotic recombination leading to cri-du-chat syndrome. *Cytogenet. Cell Genet.* **47:** 5–7

Dumont BL & Payseur BA (2008) Evolution of the Genomic Rate of Recombination in Mammals. *Evolution* **62:** 276–294

Egel R (2004) The Molecular Biology of Schizosaccharomyces pombe: Genetics, Genomics and Beyond Springer

Feng L, Lintula S, Ho TH, Anastasina M, Paju A, Haglund C, Stenman U-H, Hotakainen K, Orpana A, Kainov D & Stenman J (2012) Technique for strand-specific gene-expression analysis and monitoring of primer-independent cDNA synthesis in reverse transcription. *BioTechniques* **52:** 263–270

Gullerova M, Moazed D & Proudfoot NJ (2011) Autoregulation of convergent RNAi genes in fission yeast. *Genes Dev.* **25:** 556–568

Gullerova M & Proudfoot NJ (2008) Cohesin Complex Promotes Transcriptional Termination between Convergent Genes in S. pombe. *Cell* **132:** 983–995

Haddad F, Qin AX, Giger JM, Guo H & Baldwin KM (2007) Potential pitfalls in the accuracy of analysis of natural sense-antisense RNA pairs by reverse transcription-PCR. *BMC Biotechnol.* **7:** 21

Hurst LD, Pál C & Lercher MJ (2004) The evolutionary dynamics of eukaryotic gene order. *Nat. Rev. Genet.* **5:** 299–310

Kaiser P (1984) Pericentric inversions. Problems and significance for clinical genetics. *Hum. Genet.* **68:** 1–47

Levy B, Dunn TM, Kern JH, Hirschhorn K & Kardon NB (2002) Delineation of the dup5q phenotype by molecular cytogenetic analysis in a patient with dup5q/del 5p (cri du chat). *Am. J. Med. Genet.* **108:** 192–197

Li X-Q & Du D (2012) Gene direction in living organisms. *Sci. Rep.* **2:** Available at: http://www.nature.com/srep/2012/121221/srep00982/full/srep00982.html [Accessed May 21, 2013]

Mancera E, Bourgon R, Brozzi A, Huber W & Steinmetz LM (2008) High-resolution mapping of meiotic crossovers and non-crossovers in yeast. *Nature* **454:** 479–485

Martin RH (1991) Cytogenetic analysis of sperm from a man heterozygous for a pericentric inversion, inv (3) (p25q21). *Am. J. Hum. Genet.* **48:** 856–861

Mata J (2013) Genome-wide mapping of polyadenylation sites in fission yeast reveals widespread alternative polyadenylation. *RNA Biol.* **10:** 1407–1414

Michalak P (2008) Coexpression, coregulation, and cofunctionality of neighboring genes in eukaryotic genomes. *Genomics* **91:** 243–248

Morel F, Laudier B, Guérif F, Couet ML, Royère D, Roux C, Bresson JL, Amice V, De Braekeleer M & Douet-Guilbert N (2007) Meiotic segregation analysis in spermatozoa of pericentric inversion carriers using fluorescence in-situ hybridization. *Hum. Reprod. Oxf. Engl.* **22:** 136–141

Ni T, Tu K, Wang Z, Song S, Wu H, Xie B, Scott KC, Grewal SI, Gao Y & Zhu J (2010) The Prevalence and Regulation of Antisense Transcripts in Schizosaccharomyces pombe. *PLoS ONE* **5:** e15271

Pelechano V & Steinmetz LM (2013) Gene regulation by antisense transcription. *Nat. Rev. Genet.* **14:** 880–893

Schlackow M, Marguerat S, Proudfoot NJ, Bähler J, Erban R & Gullerova M (2013) Genome-wide analysis of poly(A) site selection in Schizosaccharomyces pombe. *RNA N. Y. N* **19:** 1617–1631

Sinha H, David L, Pascon RC, Clauder-Münster S, Krishnakumar S, Nguyen M, Shi G, Dean J, Davis RW, Oefner PJ, McCusker JH & Steinmetz LM (2008) Sequential Elimination of Major-Effect Contributors Identifies Additional Quantitative Trait Loci Conditioning High-Temperature Growth in Yeast. *Genetics* **180:** 1661–1670

Tarazona S, García-Alcalde F, Dopazo J, Ferrer A & Conesa A (2011) Differential expression in RNA-seq: A matter of depth. *Genome Res.* Available at: http://genome.cshlp.org/content/early/2011/09/07/gr.124321.111 [Accessed March 11, 2013]

Toung JM, Morley M, Li M & Cheung VG (2011) RNA-sequence analysis of human B-cells. *Genome Res.* **21:** 991–998

Wilhelm BT, Marguerat S, Watt S, Schubert F, Wood V, Goodhead I, Penkett CJ, Rogers J & Bähler J (2008) Dynamic repertoire of a eukaryotic transcriptome surveyed at single-nucleotide resolution. *Nature* **453:** 1239–1243

Williams RW, Gu J, Qi S & Lu L (2001) The genetic structure of recombinant inbred mice: high-resolution consensus maps for complex trait analysis. *Genome Biol.* **2:** research0046.1–research0046.18

Supplementary Table S1: Strains of the recombinant library studied

strain name	type	number of biological replicates studied
768	parent	7
Y0036	parent	3
R1-1	F1 recombinant	3
R1-2	F1 recombinant	1
R1-3	F1 recombinant	3
R1-4	F1 recombinant	2
R1-5	F1 recombinant	2
R1-6	F1 recombinant	2
R1-7	F1 recombinant	2
R1-8	F1 recombinant	2
R1-9	F1 recombinant	2
R1-10	F1 recombinant	2
R1-11	F2 recombinant	1
R1-12	F2 recombinant	1
R1-13	F2 recombinant	1
R1-14	F2 recombinant	1
R1-15	F2 recombinant	1
R1-16	F2 recombinant	1
R1-17	F2 recombinant	1
R1-18	F2 recombinant	1
R1-19	F2 recombinant	1
R1-20	F2 recombinant	1
R1-21	F2 recombinant	1
R1-22	F2 recombinant	1
R1-23	F2 recombinant	1
R1-24	F2 recombinant	1
R1-25	F2 recombinant	1
R1-26	F2 recombinant	1
R1-27	F2 recombinant	1
R1-28	F2 recombinant	1
R1-29	F2 recombinant	1
R1-30	F2 recombinant	1
R1-31	F2 recombinant	1
R1-32	F2 recombinant	1
R1-33	F2 recombinant	1
R1-35	F2 recombinant	1
R1-36	F2 recombinant	1
R1-37	F2 recombinant	1
R1-38	F2 recombinant	1
R1-39	F2 recombinant	1
R1-40	F2 recombinant	1
R1-41	F2 recombinant	1
R1-42	F2 recombinant	1
R1-43	F2 recombinant	1
R1-46	F2 recombinant	1
R1-47	F2 recombinant	1

Supplementary Table S2: RNA-seq summary statistics

sample	strain	total number of read	number of aligned read	proportion of aligned read	effective coverage (in number of genomes)
sample_01	R1_1	19,306,310	8,785,591	45.50%	33.5 x
sample_02	R1_2	15,094,922	6,092,214	40.40%	23.2 x
sample_03	R1_4	16,359,130	8,653,446	52.90%	33.0 x
sample_04	R1_3	16,622,097	8,506,874	51.20%	32.4 x
sample_05	R1_5	15,516,353	6,196,508	39.90%	23.6 x
sample_06	R1_6	26,935,992	11,303,550	42.00%	43.1 x
sample_07	R1_7	32,189,715	11,924,765	37.00%	45.5 x
sample_08	R1_8	21,314,940	10,500,206	49.30%	40.0 x
sample_09	R1_9	30,415,989	14,315,218	47.10%	54.6 x
sample_10	R1_10	21,214,988	10,644,620	50.20%	40.6 x
sample_11	R1_1	13,073,660	4,177,279	32.00%	15.9 x
sample_12	968	29,454,427	8,524,431	28.90%	32.5 x
sample_13	968	27,764,673	8,979,747	32.30%	34.2 x
sample_14	968	43,415,396	13,336,524	30.70%	50.8 x
sample_15	Y0036	24,881,565	8,996,992	36.20%	34.3 x
sample_16	Y0036	24,002,818	7,144,312	29.80%	27.2 x
sample_17	R1_11	23,896,327	13,125,382	54.90%	50.0 x
sample_18	R1_12	13,165,684	7,805,017	59.30%	29.8 x
sample_19	R1_13	76,435,640	38,213,066	50.00%	145.7 x
sample_20	R1_15	20,067,610	10,443,167	52.00%	39.8 x
sample_21	R1_16	9,948,690	4,997,422	50.20%	19.1 x
sample_22	R1_17	8,136,812	4,488,751	55.20%	17.1 x
sample_23	R1_18	11,131,746	6,826,220	61.30%	26.0 x
sample_24	R1_23	55,199,621	21,853,723	39.60%	83.3 x
sample_25	R1_24	11,528,050	6,453,958	56.00%	24.6 x
sample_26	R1_25	20,832,734	10,417,794	50.00%	39.7 x
sample_27	R1_26	21,328,683	10,594,453	49.70%	40.4 x
sample_28	R1_27	27,297,118	13,448,644	49.30%	51.3 x
sample_29	R1_29	29,810,913	13,858,822	46.50%	52.8 x
sample_30	R1_30	24,045,882	13,101,833	54.50%	49.9 x
sample_31	R1_31	26,309,553	13,318,957	50.60%	50.8 x
sample_32	R1_32	33,281,020	15,222,567	45.70%	58.0 x
sample_33	R1_35	19,628,725	8,988,877	45.80%	34.3 x
sample_34	R1_36	32,294,885	15,969,076	49.40%	60.9 x
sample_35	R1_37	20,925,865	10,530,042	50.30%	40.1 x
sample_36	R1_38	21,503,226	11,509,469	53.50%	43.9 x
sample_37	R1_39	24,228,075	12,558,886	51.80%	47.9 x
sample_38	R1_40	6,252,759	3,354,000	53.60%	12.8 x
sample_39	R1_41	15,838,258	7,653,585	48.30%	29.2 x
sample_40	R1_43	17,807,896	8,016,085	45.00%	30.6 x
sample_41	R1_46	24,524,432	12,962,183	52.90%	49.4 x
sample_42	R1_47	19,156,437	10,600,847	55.30%	40.4 x
sample_43	R1_28	13,262,208	5,866,689	44.20%	22.4 x
sample_44	R1_33	22,771,249	9,494,978	41.70%	36.2 x
sample_45	R1_42	11,814,312	5,565,702	47.10%	21.2 x
sample_46	Y0036	28,413,418	10,493,753	36.90%	40.0 x
sample_47	968	23,306,017	10,350,162	44.40%	39.5 x
sample_48	968	22,609,653	5,130,589	22.70%	19.6 x
sample_49	968	26,830,942	14,572,428	54.30%	55.6 x
sample_50	968	23,950,176	7,126,051	29.80%	27.2 x
sample_51	R1_19	18,575,119	8,592,677	46.30%	32.8 x
sample_52	R1_20	14,464,743	5,966,934	41.30%	22.7 x
sample_53	R1_21	16,054,540	7,020,888	43.70%	26.8 x
sample_54	R1_22	18,751,927	6,670,163	35.60%	25.4 x
sample_55	R1_14	20,901,719	8,559,869	41.00%	32.6 x
sample_56	R1_1	19,911,458	8,260,219	41.50%	31.5 x
sample_57	R1_3	23,220,070	6,384,577	27.50%	24.3 x
sample_58	R1_3	20,893,737	8,828,131	42.30%	33.7 x
sample_59	R1_4	22,467,780	10,873,138	48.40%	41.5 x
sample_60	R1_5	22,647,687	10,190,052	45.00%	38.8 x
sample_61	R1_6	21,809,157	9,728,376	44.60%	37.1 x
sample_62	R1_7	18,790,329	8,211,097	43.70%	31.3 x
sample_63	R1_8	18,305,899	7,921,197	43.30%	30.2 x
sample_64	R1_9	17,839,600	7,588,315	42.50%	28.9 x
sample_65	R1_10	25,507,656	10,595,067	41.50%	40.4 x
sample_66	swc5_del	17,517,012	6,626,954	37.80%	25.3 x
sample_67	swc5_del	21,493,905	7,994,691	37.20%	30.5 x
sample_68	swc5_del	17,049,177	6,299,497	36.90%	24.0 x

Supplementary Table S3: Description of the eQTL hotspot

hotspot	Position(kb)	Number of sense traits (antisense traits)	Strongest candidate regulator	shared function among targets$^{\Omega}$
1	I:522-578	25 (3)	SPAC2F7.02c	ribosome biogenesis (92%)
2*	I:2666-3130	400 (177)	SPAC1B2.06	response to stress (33%)
3*	I:3307-3745	583 (162)	SPAC1B1.02c	-
4	I:3816-3917	67 (13)	SPNCRNA.944	-
5*	I:4611-4947	159 (76)	SPNCRNA.1018	response to stress (40%)
6	I:5402-5454	26 (12)	SPNCRNA.28	reponse to starvation (20%)
7	III:657-708	72 (6)	SPCC16C4.06c	cell wall (47%)
8*	III:2081-2123	871 (1,390)	SPCC576.13	-

* Significantly enriched for antisense linkage (if the eQTL were randomly distributed across the genome, no bins should contain more than 21 eQTLs, or 24 aseQTLs; all the hotspot described here are enriched for eQTLs)

Ω The shared function has been determined by GO enrichment analysis and is idicated when it is shared by more than 10% of the targets (**Supplementary Table S4**)

Supplementary Table E4: GO enrichments for each 8 hotspots. Only significant terms (p <0.005) are indicated.

	GO identification	Term	p.value
Hotspot 1			
	GO:0042254	ribosome biogenesis	1.80E-24
Hotspot 2			
	GO:0006950	response to stress	0.00047
	GO:0000296	spermine transport	0.00078
	GO:0015848	spermidine transport	0.00078
	GO:0042327	positive regulation of phosphorylation	0.0056
	GO:0090028	positive regulation of pheromone-dependent	0.0056
	GO:0006835	dicarboxylic acid transport	0.0056
	GO:0071294	cellular response to zinc ion	0.0056
	GO:0007329	positive regulation of transcription fro...	0.0056
Hotspot 3			
	GO:0006882	cellular zinc ion homeostasis	0.00037
	GO:0071471	cellular response to non-ionic osmotic stress	0.00145
	GO:0071475	cellular hyperosmotic salinity response	0.00145
	GO:0009247	glycolipid biosynthetic process	0.00171
	GO:0000032	cell wall mannoprotein biosynthetic process	0.0026
	GO:0043940	regulation of sexual sporulation	0.00344
	GO:0000752	agglutination involved in conjugation	0.00344
	GO:0006497	protein lipidation	0.00497
Hotspot 4			
	GO:0006535	cysteine biosynthetic process from serine	0.00047
	GO:0030100	regulation of endocytosis	0.0058
Hotspot 5			
	GO:0015848	spermidine transport	0.000048
	GO:0000296	spermine transport	0.000048
	GO:0006950	response to stress	0.00059
	GO:0006835	dicarboxylic acid transport	0.00087
Hotspot 6			
	GO:0009267	cellular response to starvation	0.00025
	GO:0006301	postreplication repair	0.00092
Hotspot 7			
	GO:0071554	cell wall organization or biogenesis	1.20E-11
	GO:0051274	beta-glucan biosynthetic process	0.000011
	GO:0010981	regulation of cell wall macromolecule	0.000026
	GO:0044275	cellular carbohydrate catabolic process	0.0001
	GO:2000769	regulation of establishment or maintenance of cell polarity regulating cell shape	0.00035
	GO:2000021	regulation of ion homeostasis	0.00037
	GO:0060402	calcium ion transport into cytosol	0.00037
	GO:0046379	extracellular polysaccharide metabolic process	0.00044
	GO:0051666	actin cortical patch localization	0.00044
	GO:0060341	regulation of cellular localization	5.50E-04
	GO:0010959	regulation of metal ion transport	0.00182
	GO:0034975	protein folding in endoplasmic reticulum	0.00335
	GO:0032319	regulation of Rho GTPase activity	0.00427
	GO:0009101	glycoprotein biosynthetic process	0.00446
	GO:0008360	regulation of cell shape	0.00669
	GO:0071472	cellular response to salt stress	0.00899
Hotspot 8			
	GO:0006359	regulation of transcription from RNA polymerase II	0.0016
	GO:0006839	mitochondrial transport	0.0028
	GO:0006370	7-methylguanosine mRNA capping	0.0059
	GO:0042255	ribosome assembly	0.0059

Supplementary Table S5: GO enrichment of the *swc5* eQTL targets up-regulated in *swc5-fs* strains.

GO identification	Term	p.value
GO:0033554	cellular response to stress	6.90E-03
GO:0051156	glucose 6-phosphate metabolic process	0.0079
GO:0042138	meiotic DNA double-strand break formation	0.0079

Supplementary Table S6: Primers used for the qPCR experiments

**Sense RT primers**	
its3-SRT-2	CGGTGTTCTGTTTTGGGTGA
tpp1-SRT-2	GCCAAATCAGCAAGGATATCAA
gpi3-SRT-2	CCGTTAATACCAGAGATTCGTCA
lsm1-SRT-2	ATGTCCGCCGTCAACACTAA
paf1-SRT	TCTTCTACTGGTGGTGCAGG
rps1602-SRT	TCCATACGACGAGGATCAGC
spac29e6.09-SRT-2	CTCGATACATGAATCACCGTCA
spac29e6.10c-SRT	TGTCCAAGTACGATTTCCCCA
thi4-SRT-2	AGTACCAGGACCTTGAACGAG
cdb4-SRT-2	GGAGAGCCACAATTTCTGGG
spbc1709.14-SRT	ACATTCCCCGTTTGTCTTGC
ctf2-SRT	TTGCTCAAACTGCCCTCAAC
act1-SRT-2	AGGGAGGAAGATTGAGCAGC
cdc2-SRT	GGGCAGGGTCATAAACAAGC

**Antisense RT primers**	
its3-ASRT	CCACCTGCTGAATACGCTTC
gpi3-ASRT	GTCGCGGAAAGGACAGAAAA
paf1-ASRT-2	CTCGTTCTACCCTAAAGCGC
spac29e6.10c-ASRT	AAAGGATGGGTAGTCGTGCA
cdb4-ASRT-2	TGAAGCGTTCTGACATTGCT
ctf2-ASRT	TGCTGGCTAAGGTTGAGATCT
act1-SRT-2	AGGGAGGAAGATTGAGCAGC
cdc2-SRT	GGGCAGGGTCATAAACAAGC

**qPCR primers**	
tpp1-qpcr1-lo	CTTGAAGTGCGTCCTTCCAG
tpp1-qpcr1-up	GGGTCTGCGATACACCAGT
its3-qpcr1-lo	TTGCGAGAAGAAGGAGCTGT
its3-qpcr1-up	GATGGCCAGCGTGTTAACAA
lsm1-qpcr1-lo	ACAATTGCGACGTATGCCTG
lsm1-qpcr1-up	GGTACTTGCCCTTCTCACGT
gpi3-qpcr1-lo	CGCTGGGTCAATATCAGATGC
gpi3-qpcr1-up	ATTACGGATGTGGGCAATGG
rps1602-qpcr1-lo	GTTTCTGGTGGTGGACATGT
rps1602-qpcr1-up	AGCCTTCTTGAGTTCAGCCT
paf1-qpcr1-lo	GCCGACATATTCTGAGCTTGT
paf1-qpcr1-up	CTGAAGAAGAGAAACCTGCGG
spac29e6.10c-qpcr1-lo	TCACAGCTTCTAATTCAACCACT
spac29e6.10c-qpcr1-up	GTCGACCGAGATATTTTCCAGG
spac29e6.09-qpcr2-lo	ATGTGATGAAGCGCCATTCC
spac29e6.09-qpcr2-up	GCTATAGACACTGCTGAGCG
cdb4-qpcr1-lo	GCTCGATAAAGACGGAGGCA
cdb4-qpcr1-up	TCTTCCTTAGGTTCAGAATCGC
thi4-qpcr1-lo	TCGCTTCCACTGCTAATTCA
thi4-qpcr1-up	TGATGGGTGTAACGGCTTCT

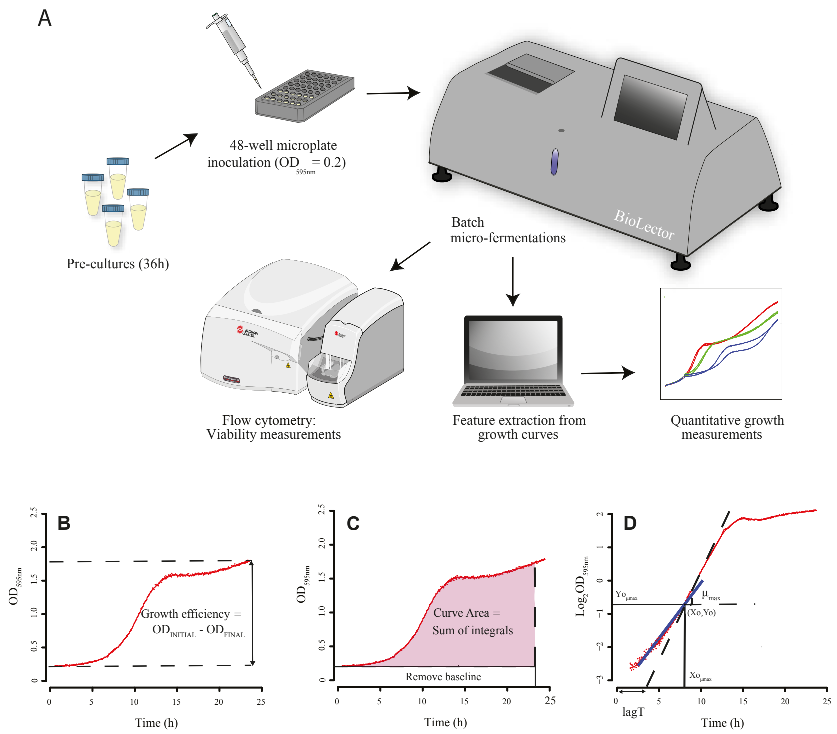

Supplementary Figure S1. Growth characterization of the recombinant strain library.

A. Experimental procedure. A 48-well microtiter plate, which is compatible with the BioLector micro-fermentation system, is inoculated at a starting optical density (OD_{295nm}) of 0.2 using cells grown in pre-cultures for 36 hours. The plate is then sealed with gas-permeable membranes and loaded in the BioLector system, and the desired experimental conditions are chosen. After the end of a BioLector experiment, growth data are collected from the system and an aliquot of each culture is analyzed for cell viability using the Cell Lab Quanta SC MPL flow cytometer. The growth profiles recorded by BioLector are then retrieved and the growth data is analyzed to extract the quantitative measures that precisely describe the growth of the cultivated strains.

B. Growth efficiency is the gain of biomass provided by the given the nutrients in the culture and is calculated by subtracting the optical density of the culture at the stationary phase of growth from the optical density at the time of inoculation.

C. An indicator of growth is the area under the growth curve, which is calculated from the sum of integrals.

D. When log-transformed OD values are plotted over time, the maximum growth rate (μmax) corresponds to the maximum slope within the exponential growth limit and the lag time (lagT) is the time at which the tangent to the maximal growth rate intersects the horizontal axis. The growth amplitude (Yo, μmax) and the time (Xo, μmax) when cells reach the μmax are also useful indicators of cellular growth.

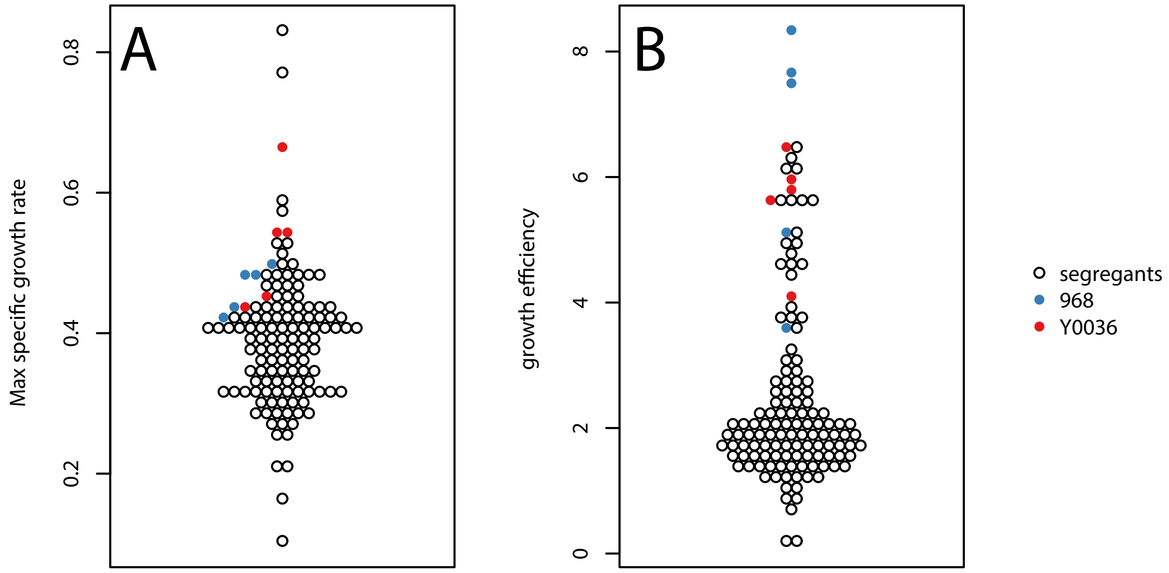

Supplementary Figure S2. Maximum specific growth rate (A) and growth efficiency (B) in the strain library.

Each point corresponds to a biological replicate of the measurements. The parental strains show higher Maximum specific growth rate and growth efficiency than most of the segregants.

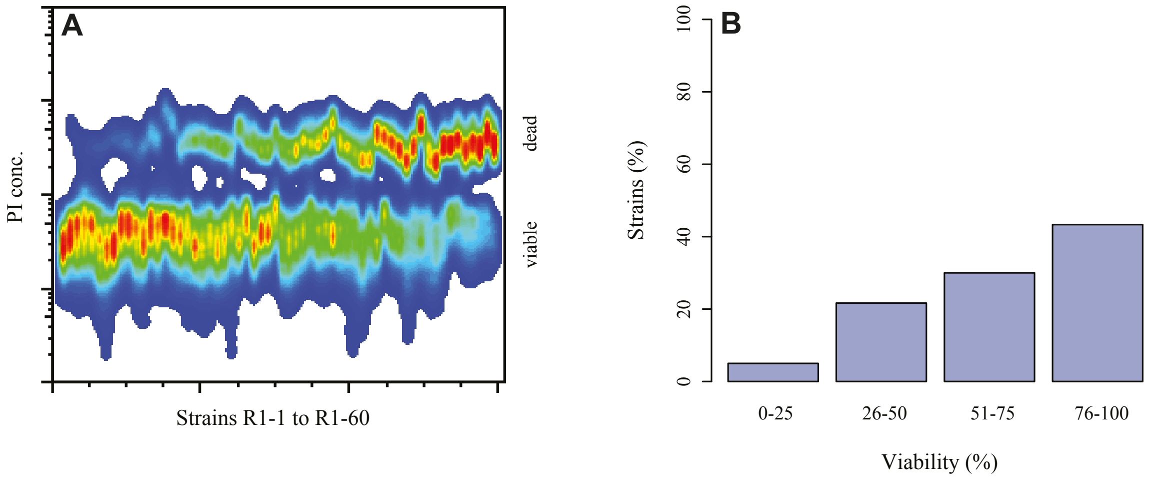

Supplementary Figure S3. Cell survival at the stationary phase of growth for all segregant strains.

A. Survival plot showing the density of live and dead cells from cultures that were grown for 36 hours. Cells were separated in two groups (live and dead) according to the concentration of propidium iodide (PI) they absorbed. Increasing cell density in either group is indicated by a color gradient, where the dark blue color indicates low and the red color a high cell density.

B. Fraction of segregant strains that showed average population viability of 0-25%, 26-50%, 51-75% and 76-100% after 36 hours in liquid cultures.

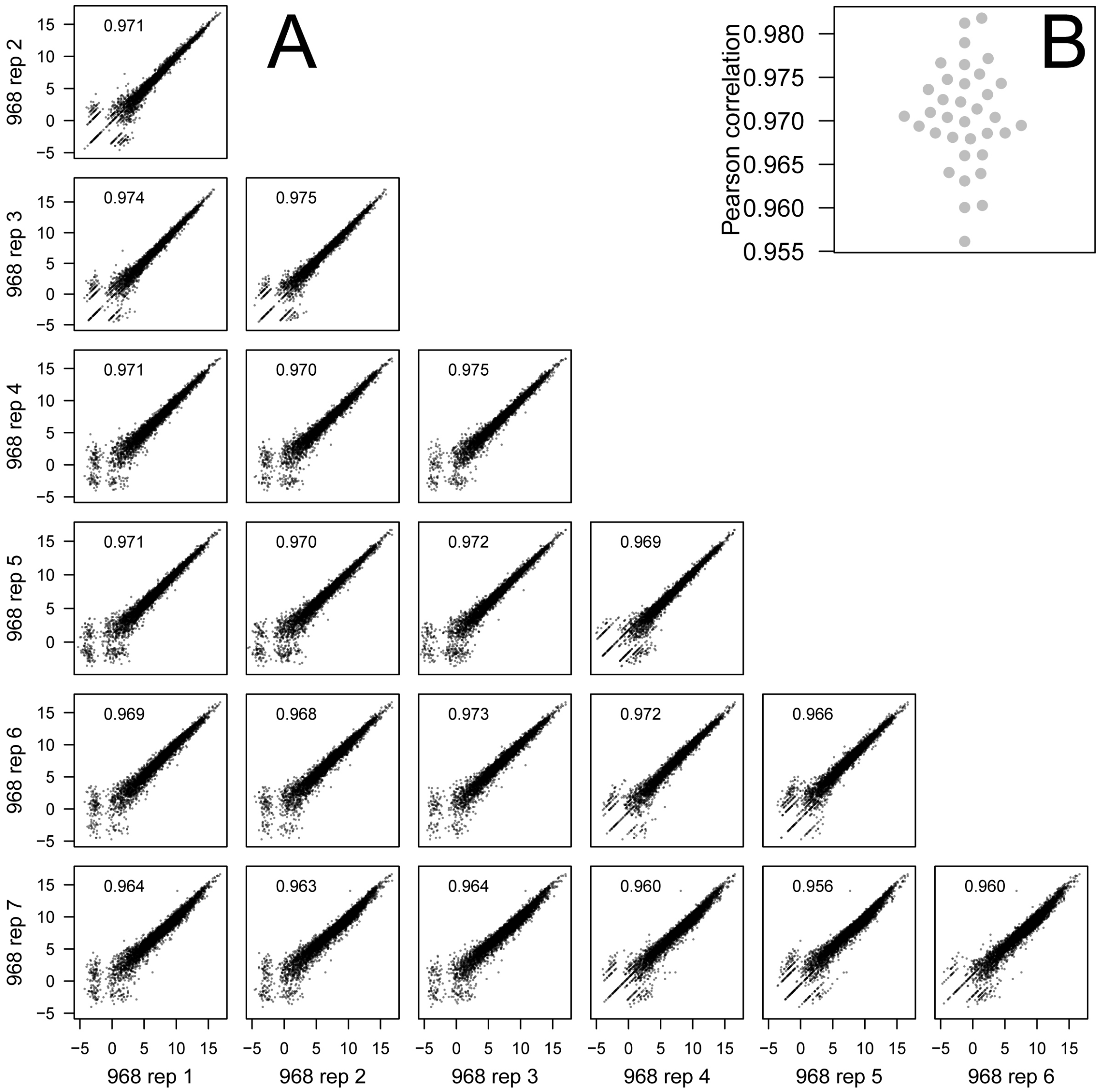

Supplementary Figure S4. Correlation between biological replicates.

A. Pairwise comparisons of the gene expression levels in between the replicates of measurements of the 968 strain. In each scatterplot the Pearson's product-moment correlation coefficient has been indicated.

B. Pearson's product-moment correlation coefficient between all the biological replicates; all the strain of the library that have been subject to replicate measurements were considered.

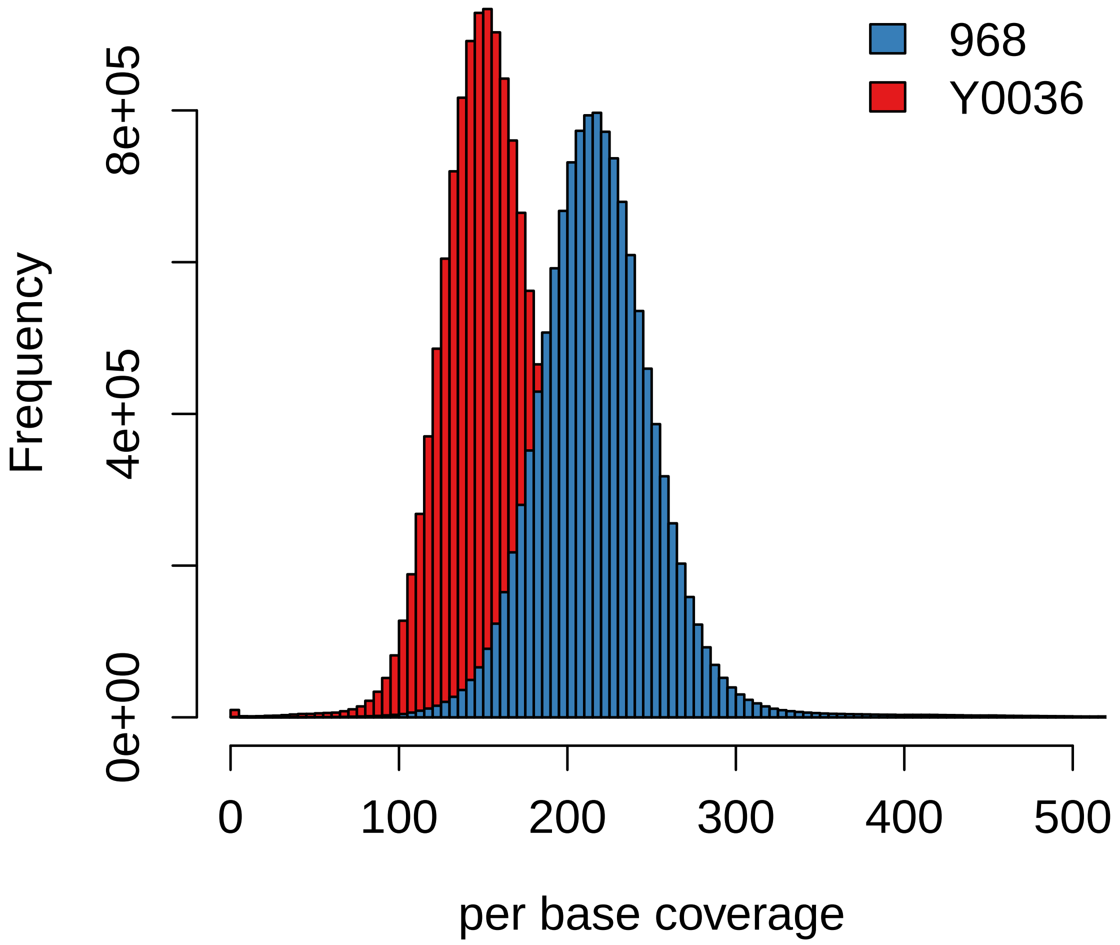

Supplementary Figure S5. Distribution of the read coverage (per base) obtained by sequencing the two parental strains. Such high sequencing depth allows to potentially detect all SNPs and small indels.

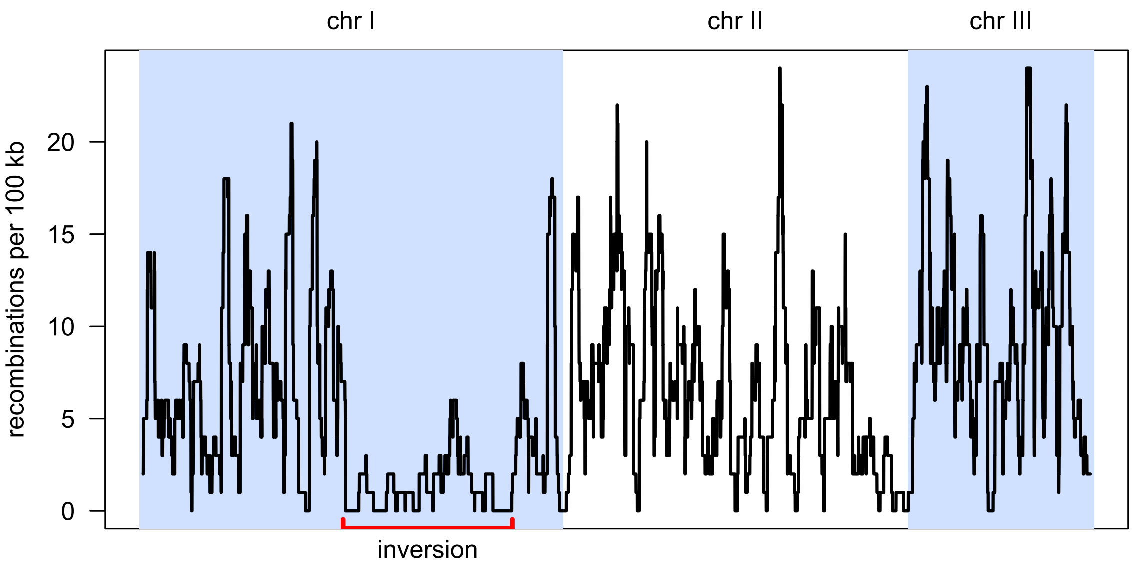

Supplementary Figure S6. Recombination landscape of the segregant library at 1kb resolution. There are fewer recombination events in a large portion of Chromosome I, corresponding to a known inversion between the two parental strains

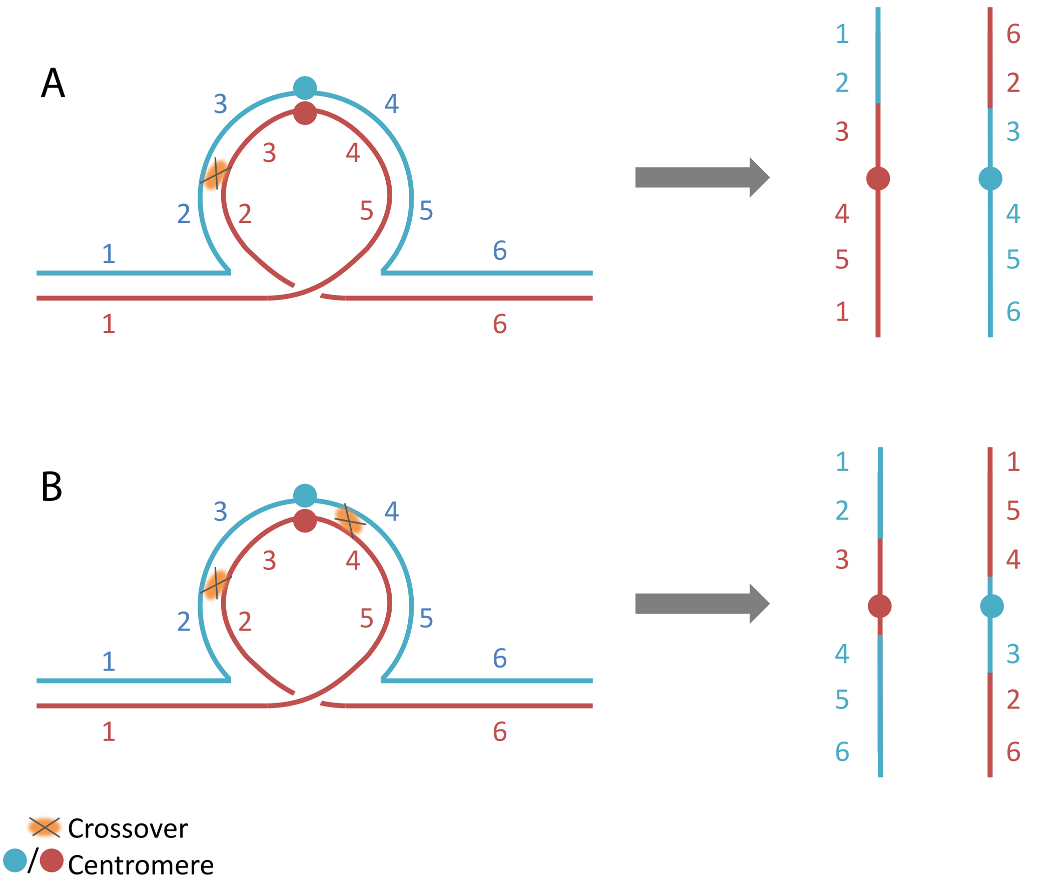

Supplementary Figure S7. Schematic representation of meiotic recombination of heterozygous pericentric inversion. For the sake of simplicity, only one copy of each homologous chromatid is represented. Pericentric inversions lead to the formation of inversion loop (twisting and folding) during the meiosis. (A) Odd number of crossovers in the inversion loop results in unbalanced chromosomes, whereas (B) even numbers result in balanced chromosomes. This complex phenomenon may explain the lack of recombination observed in the chromosome I inversion region in the strain library.

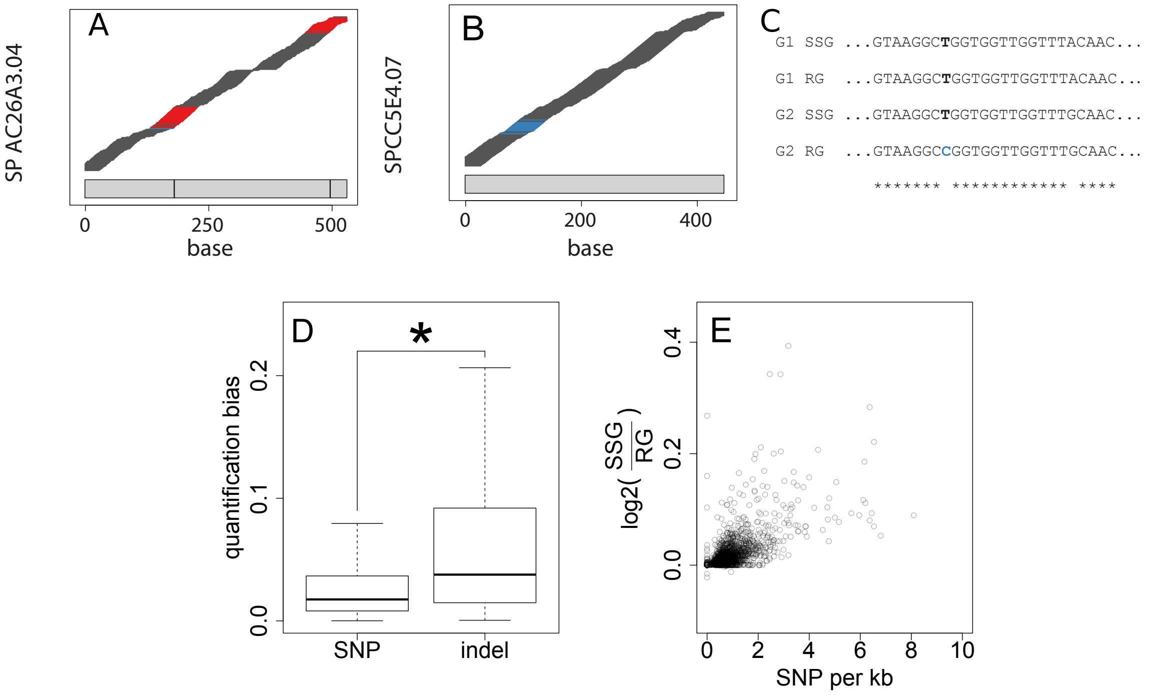

Supplementary Figure S8. Influence of polymorphisms on RNA-seq based expression quantification.

A-C. Comparative representation of RNA-seq reads mapped along selected genes when using SSG or RG mapping. In black are the reads which were mapped at the same position in both mapping methods. In red are the reads mapped only on SSG, in blue are the reads mapped only on RG. The grey box symbolizes the gene, and black vertical line the positions of the SNPs.

A. Example of a gene (SPAC26A3.04) showing an increased quantification when RNA-seq reads are mapped on SSG.

B-C. Example of a gene (G1 stands for SPCC5E4.07 on Chromosome III position 656625 to 657071) with a decreased quantification when mapped on SSG. The gene sequence is not polymorphic. However a homologous sequence on gene G2 (G2 stands for SPBC776.11 on chromosome II position 3196491 – 3196937) is polymorphic between the progenitor strains. In the Y0036 background those two sequences are identical and RNA-seq reads cannot be unambiguously mapped (C).

D. Comparison of the quantification bias in genes carrying SNPs vs indels. The quantification bias is the log ratio expression values obtained using SSG mapping over RG mapping. Only the values which were higher in SSG mapping were considered. The bias is significantly greater when genes contain indels $p < 10^{-15}$, one-sided Wilcoxon rank-sum test). The bias is also significantly greater when all measurements are considered ($p < 10^{-15}$, one-sided Wilcoxon rank-sum test).

E Correlation between the polymorphism density and the magnitude of the expression quantification bias. For each gene containing a polymorphism, the log-ratio of its average expression obtained via SSG mapping over RG mapping is plotted against its SNP density. Two points are not plotted for visual ease (SPCC1906.03 x=0, y=-0.435; SPNCRNA.1685 x=2.94, y=0.372). Both quantities are positively correlated (Spearman's $\rho = 0.90, p < 10^{-15}$).

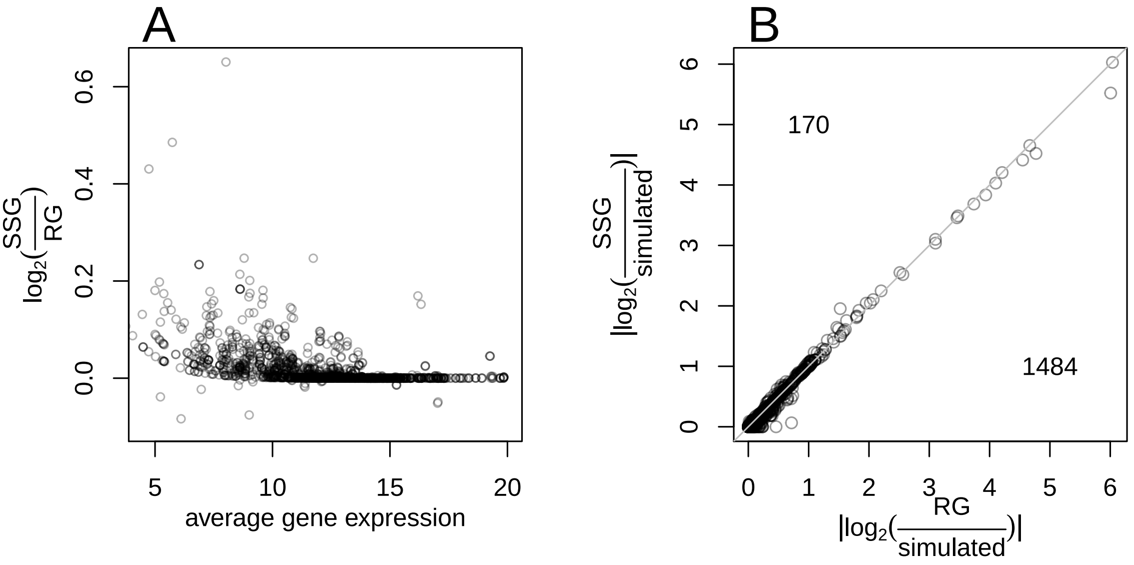

Supplementary Figure S9. Comparison of the SSG and RG mapping strategies on simulated data.

A. Comparison of average gene expression quantification obtained by SSG and RG mapping strategies in the five RNA-seq simulations. The results of the five tested simulation (corresponding to parent 968, parent Y0036, segregant R1-10, segregant R1-13, segregant R1-22) were pooled on the same plot. Only the genes which expression was differentially quantified are represented (1654 genes in total over the five simulaitons).

B. Comparison of the error of quantification when using strain-specific mapping (SSG) versus reference mapping (RG). Quantification errors were evaluated as the absolute log difference between the simulated expression levels and the measured expression levels. The number in the quadrants represent the number of points in each. In most of the case strain-specific genome mapping reduced the measurement error, but the magnitude of the improvement is small.

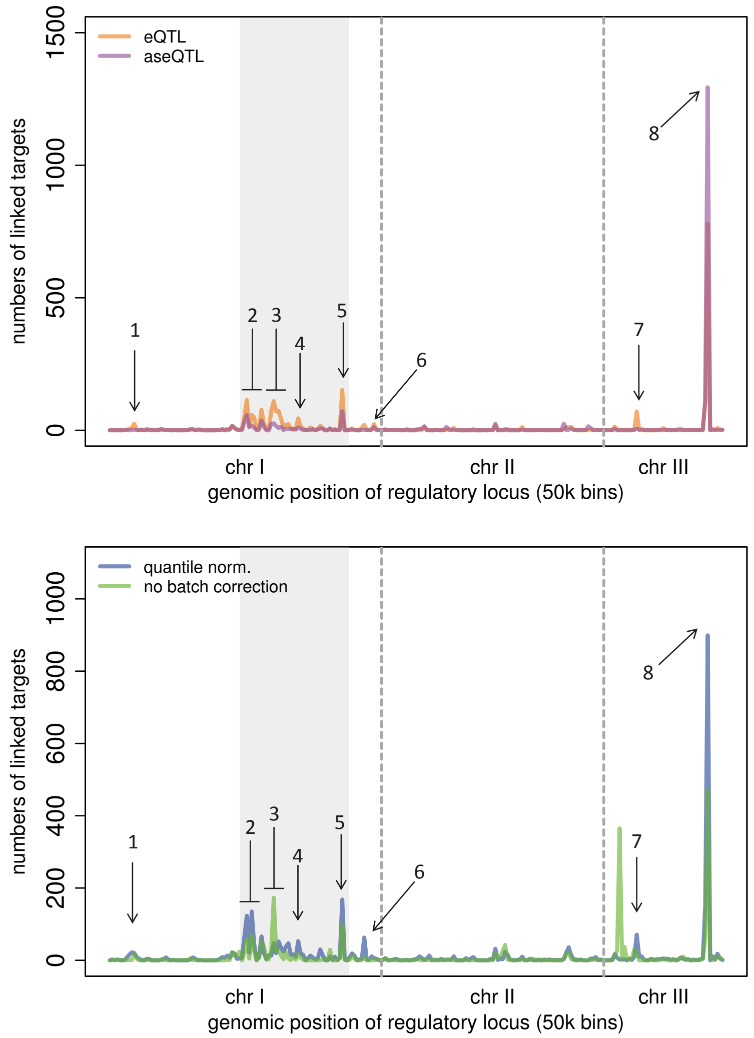

Supplementary Figure S10. eQTL and asQTL hotspots

A. Distribution of eQTLs ans asQTLs on the genome (eQTL hotspots). The genome was divided in 250 bins of 50 kb. The number of linkages falling in each bin is plotted. Dashed vertical lines indicate chromosome borders. No bins should contain more than 21 eQTLs by chance if the eQTLs were randomly distributed. The zone in grey correspond to the Chromosome I inversion. The bins showing an excess of linkage were regrouped in eight eQTL hotspot regions that were numbered from 1 to 8. The genes linked to these hotspots defined groups that were subsequently analysed (**Supplemetary Table S3**).

B. Comparison of the eQTL hotspots found when using alternative normalization strategies. In order to evaluate whether the detected hotspots were artifacts due to normalization biases, we tested other normalization strategies: the same as described (**Materials and Methods**) without batch correction (green) and the quantile normalization (Bolstad et al, 2003) (blue). eQTLs were mapped and hotspots were detected as previously described (**Materials and Methods**). Fewer eQTLs were detected when using these alternative strategies. The numbered regions correspond to the hotspots detected using the original normalization strategy. All the previously detected hotspots were also significant using both alternative normalization strategies, except for hotspots 4 and 6 that were not significant when the batch correction was not applied (green).

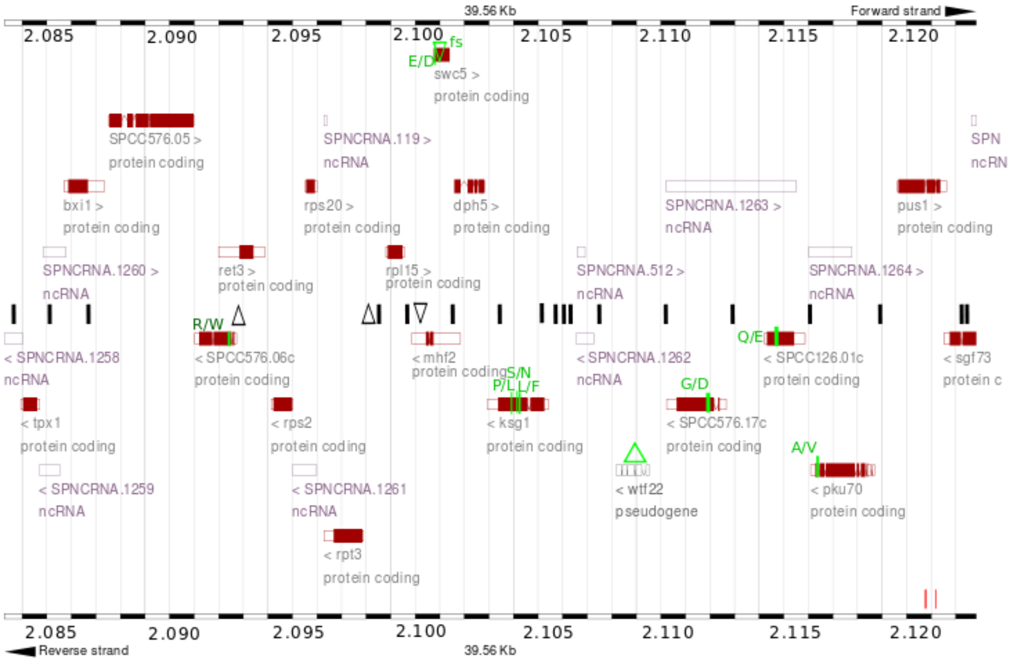

Supplementary Figure S11. Genomic map of the Chromosome III hotspot (hotspot 8).
The genomic variations between 968 and Y0036 strains (parental strains) are indicated. SNPs are indicated as vertical bars; insertions as down-pointing triangles; deletions as up-pointing triangles. Non-coding variations are colored in black and displayed in the center of the map. Non-silent polymorphisms are highlighted in green and displayed in the genes they affect. The resulting protein sequence variations are also indicated. The figure is based on the PomBase (http://www.pombase.org) genome browser (Kersey et al, 2012).

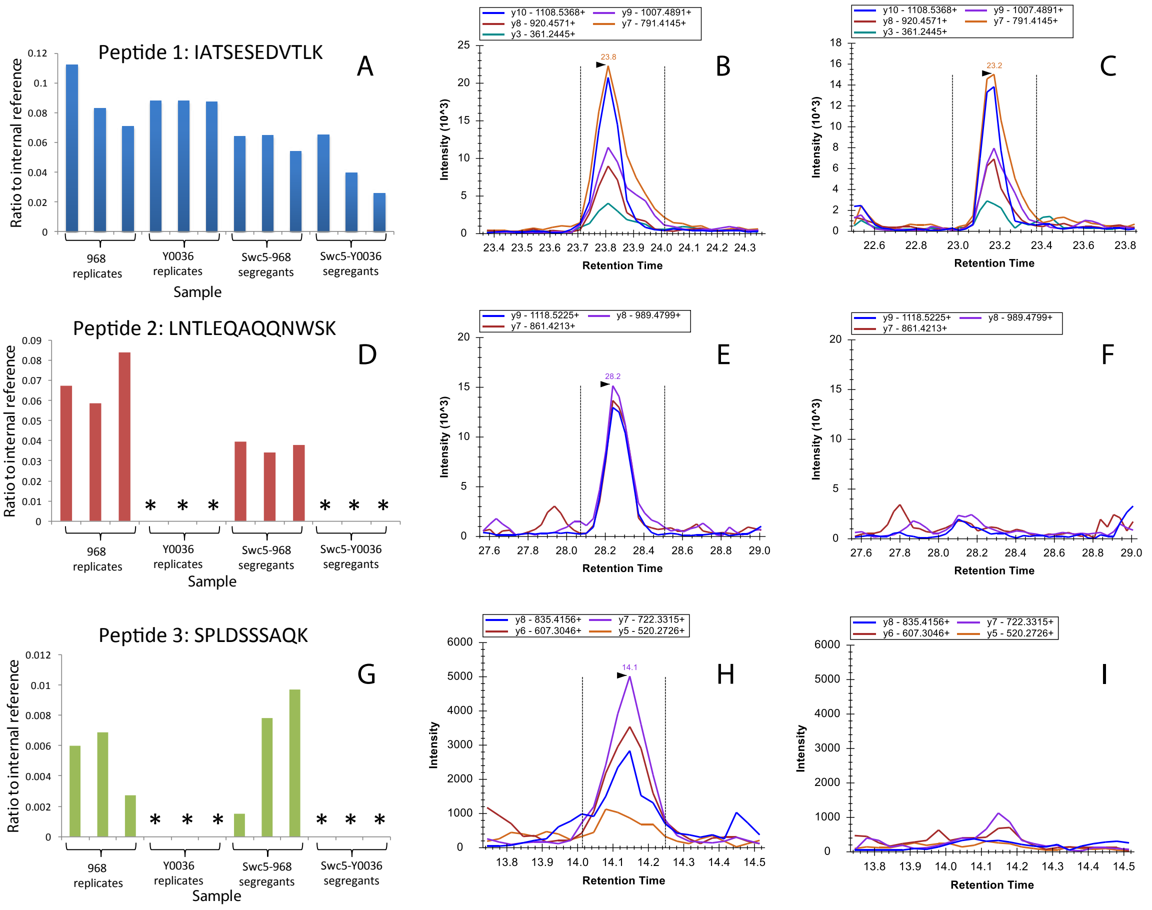

Supplementary Figure S12. Quantitative targeted proteomics analysis of Swc5.

A, D, G. The ratios of the peptide relative to spiked in aliquots of a chemically synthesized internal heavy reference peptides for all samples are shown. Peptide 1 (A) is located located before the frameshift. Petides 2 and 3 (D, G) are located after the frame shift. MS-signals with signal-to-noise ratios of less than 3 are not considered (*).

B+C, E+F, H+I. Extracted ion chromatograms (XICs) for selected peptide transitions determined by the Skyline software. XICs are shown for one 968 sample (B,E,H) and Y0036 sample (C,F,I). Traces are displayed for (B+C) peptide 1 (IATSESEDVTLK), (E+F) peptide 2 (LNTLEQAQQNWSK) and (H+I) peptide 3 (SPLDSSSAQK).

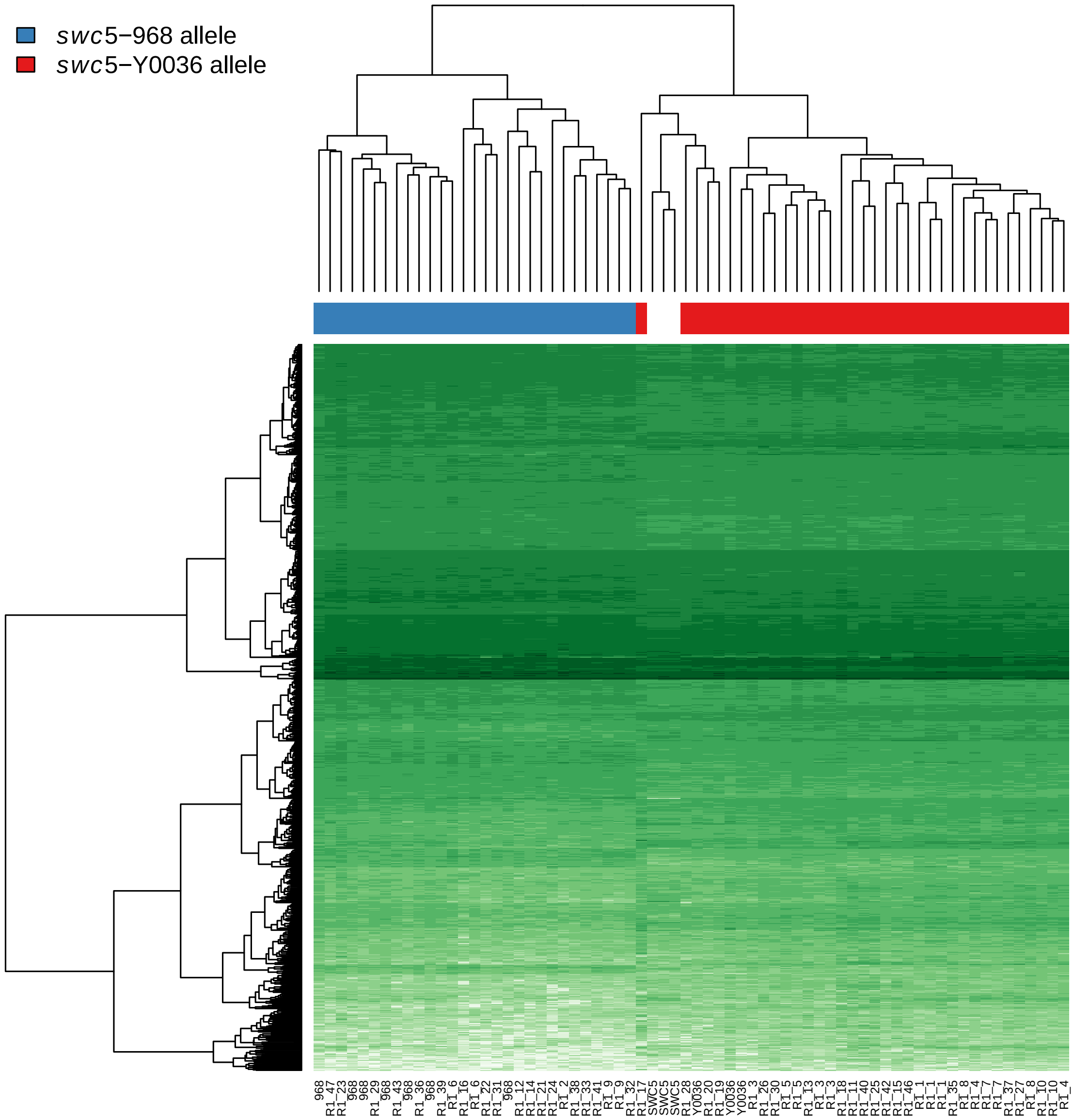

Supplementary Figure S13. Two-way cluster analysis of the expression data of the 871 gene targets of the *swc5* eQTL hotspot.

The Analysis was carried out in 68 samples derived from 44 lines from the recombinant library, the two parental strains, and the swc5 deletion strain. Columns are clustered according to the samples, and rows are clustered according to the expression. Abundance levels are color coded in a green gradient (light, low abundance; dark, high abundance). The triplicates *Δswc5* samples cluster with the strains carrying the *swc5* frame shift allele.

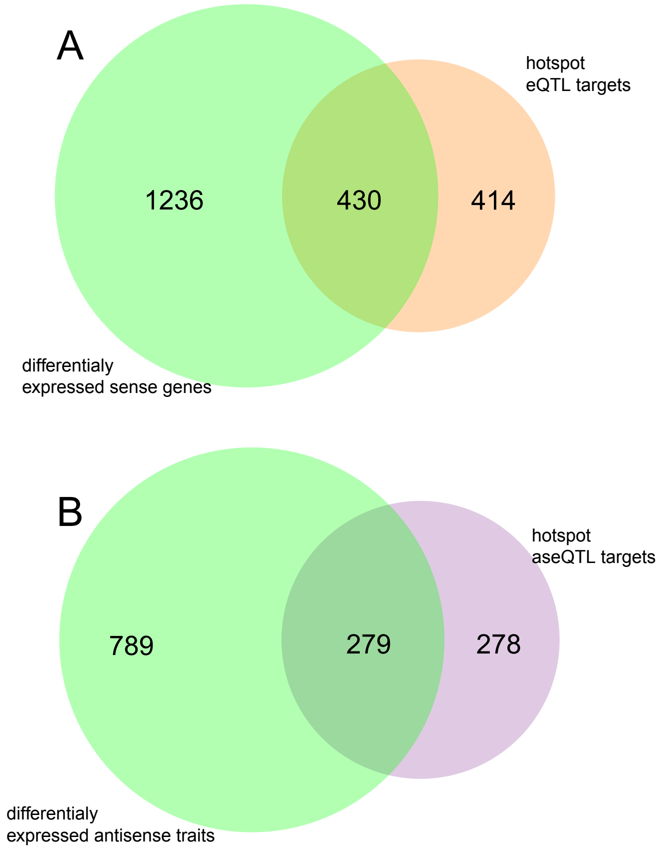

Supplementary Figure S14. Overlap between the genes differentially expressed in *Δswc5* and hotspot 8 QTL targets.

The (A) sense and (B) antisense genes differentially expressed in *swc5* deletion strain (as compared to 968) are overlapping with the targets of of the hotspot 8 (*swc5* locus). Note that only the traits that were measurable in all *Δswc5* replicates were considered.

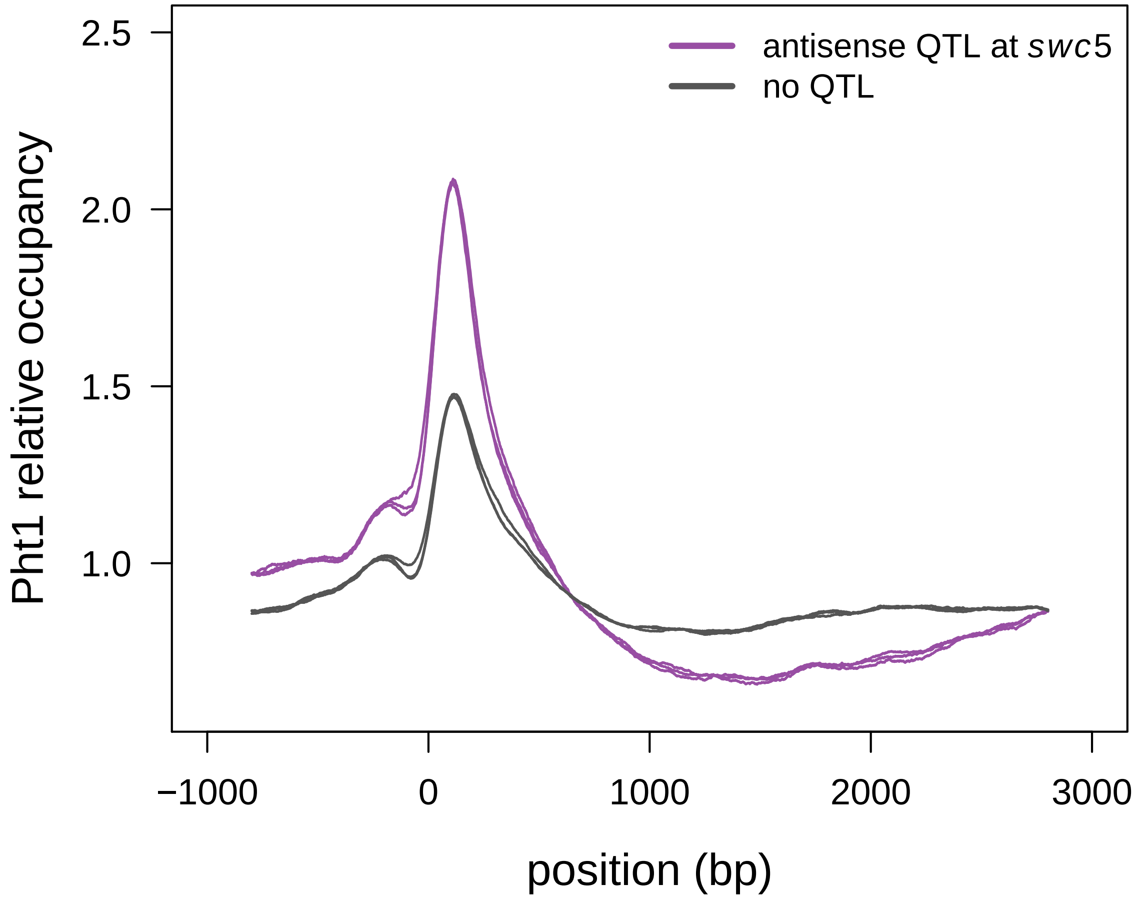

Supplementary Figure S15. Comparative average Pht1 occupancy in 968 (*scw5*+) of the genes for which antisense expression changes are linked (purple, n=1,384) and not linked (black, n=3,722) to the *swc5* locus. There is significantly more Pht1 at the +1 nucleosome in genes whose antisense level is associated with the *swc5* QTL than in the others ($p < 10^{-15}$, one sided Wilcoxon rank-sum test).

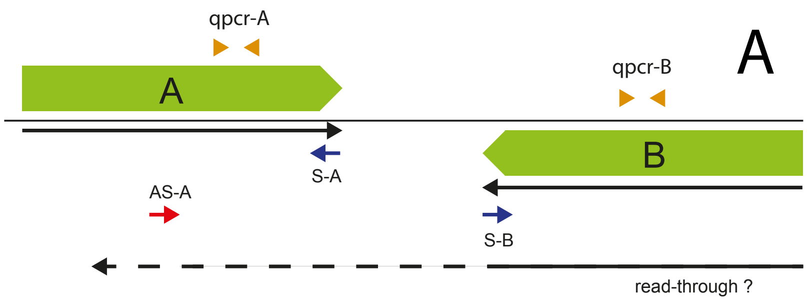

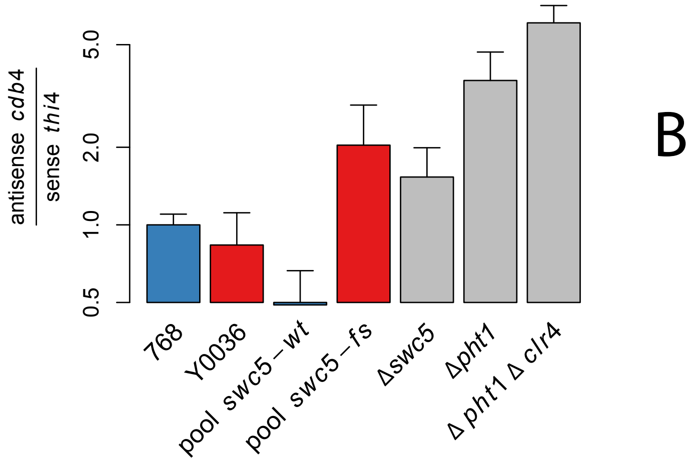

Supplementary Figure S16. RT-qPCR validation of *swc5-fs* effects.

A. Schematic representation of the design of the RT-qPCR experiments. Gene A represents the gene, which sense and antisense levels were linked to *swc5* locus. Gene B is the gene on the opposite strand arranged in a convergent way. Two RT reactions were performed for each strain interrogating the sense levels (using, the RT primers S-A and S-B, in blue, for these gene pair and the sense-RT primers of the other convergent gene pairs as well as a primer specific of *cdc2*, for normalization), and the antisense level (using, the RT primers AS-A, in red, for these gene pair and the primer of the antisense-RT primers of the other convergent gene pairs as well as a primer specific of *cdc2*, for normalization). The qPCR to assess the sense levels of gene A and gene B were done using their respective qPCR primers (qpcr-A, and qpcrB primers) and the product of the sense RT. The qPCR assessing the level of the antisense of gene A was performed using the qpcr-A primers and the product of the antisense RT. Finally read-through was assessed using the qpcr-B primers with the product of the antisense RT.

B. The increase in antisense level of *cdb4* is not due to an increase of *thi4* sense level. The 5'UTR of *thi4* overlap with the CDS of *cdb4*. Therefore the increase of *cdb4* antisense or sense level could simply be due to an increase of *thi4* sense level. Here we show the ratio of *cbd4* antisense level on *thi4* sense levels. This ratio follows the antisense-to-sense ratio of *cdb4* (**Figure 7A**). Error bars indicate the standard error to the mean among the three biological replicates.

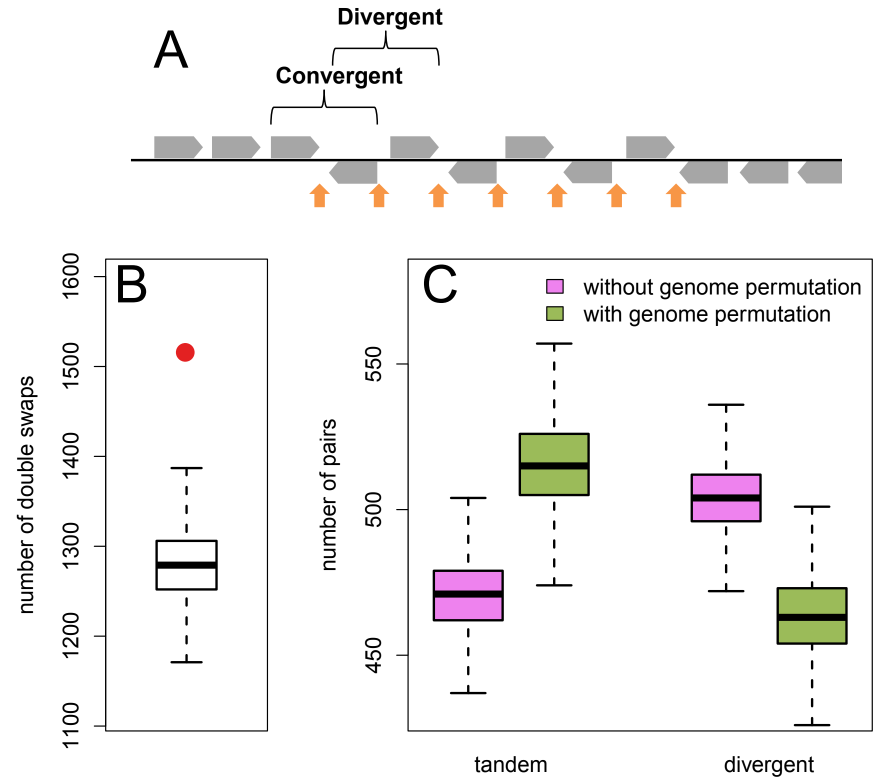

Supplementary Figure S17. The depletion of tandem genes pairs among the *swc5* aseQTL target is due to a genome organization bias.

A. Schematic representation of a stretch of convergent/divergent gene pairs. In this genome organization, when a gene of a convergent gene pair is an aseQTL target, the adjacent divergent pair is selected. Orange arrows indicate orientation swaps. Successive orientation swaps characterize convergent/divergent stretches.

B. Number of successive gene direction swaps in the fission yeast genome (red dot), and in 1,000,000 randomized genomes (boxplot). The orientation of the genes in fission yeast genome is not random.

C. Distribution of the number of adjacent tandem and divergent gene pairs selected when picking randomly aseQTL convergent pairs in the *S. pombe* genome (fuchsia), and a shuffle genome (green).

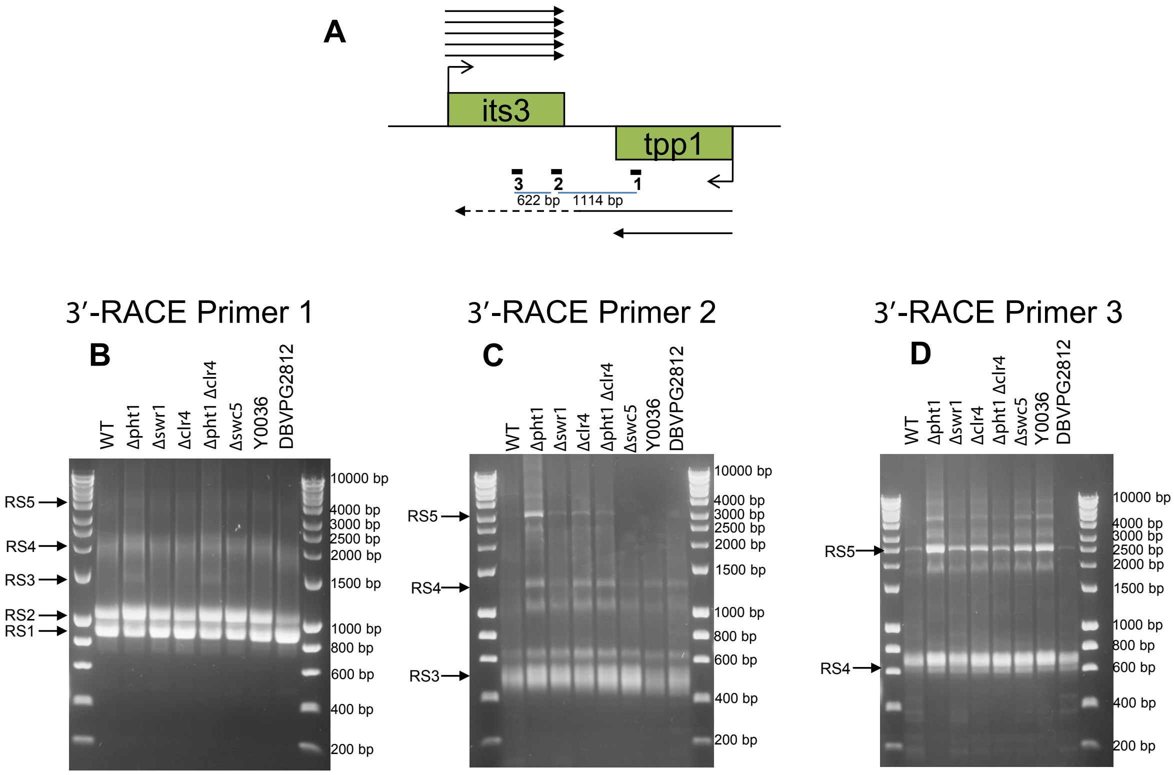

Supplementary Figure S18. 3’-RACE to detect read-through transcripts from the gene *tpp1*. Three different oligonucleotides were used to prime the 3’-RACE experiment: 3’-RACE Primer 1 (A, B), located into *tpp1* ORF; 3’-RACE Primer 2 (A, C) and 3’-RACE primer 3 (A, D), located downstream of the normal 3’ termination signal. Distance between oligonucleotides is indicated in A. At least five prominent 3’-RACE signals (RS) have been located with the three oligonucleotides used (RS1 to RS5) as seen in B, C, and D. Most *tpp1* transcripts terminate at annotated *tpp1* 3’ end (RS1 and RS2), although read-through transcripts are detected (RS3, RS4 and RS5) are detected even in wildt type (and DBVPG2812). Y0036 strain as well as other mutants known to affect transcript read-through show semi quantitative stronger bands in the 3’-RACE signals obtained downstream of the normal 3’ ends (B and C).

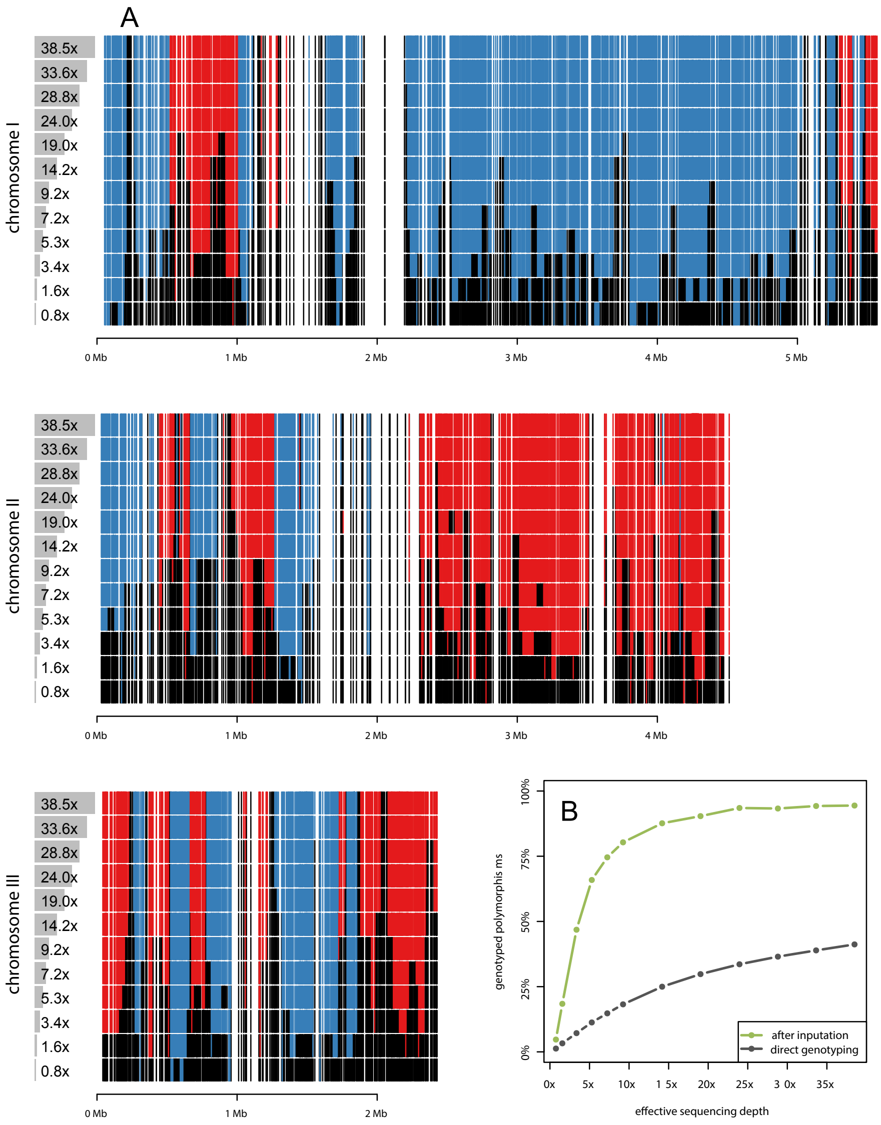

Supplementary Figure S19. Evaluation of the influence of sequencing depth on the RNA-seq based genotyping.
The reads from one sample (sample 59) were successively subset, and the genotype of strain R1-4 was inferred based on the subsets. For the sake of clarity, only one sample is shown; we obtained similar results with all the samples tested.

A. Genotype calls at 4,578 sites polymorphic between 968 and Y0036 parental strains. The grey horizontal bars represent the effective sequencing depth of the subsets (the number of mapped bases scaled to the genome size); the values are also indicated (1x correspond to one time the genome size).

B. The proportion of sites that could be genotyped in the different subsets is plotted against the effective sequencing depth. The black line represents the sites for which the genotype could be directly called. The green line corresponds to the final genotypes after the missing values were imputed from the segregation pattern of the flanking polymorphisms segregation (**Materials and Methods**).

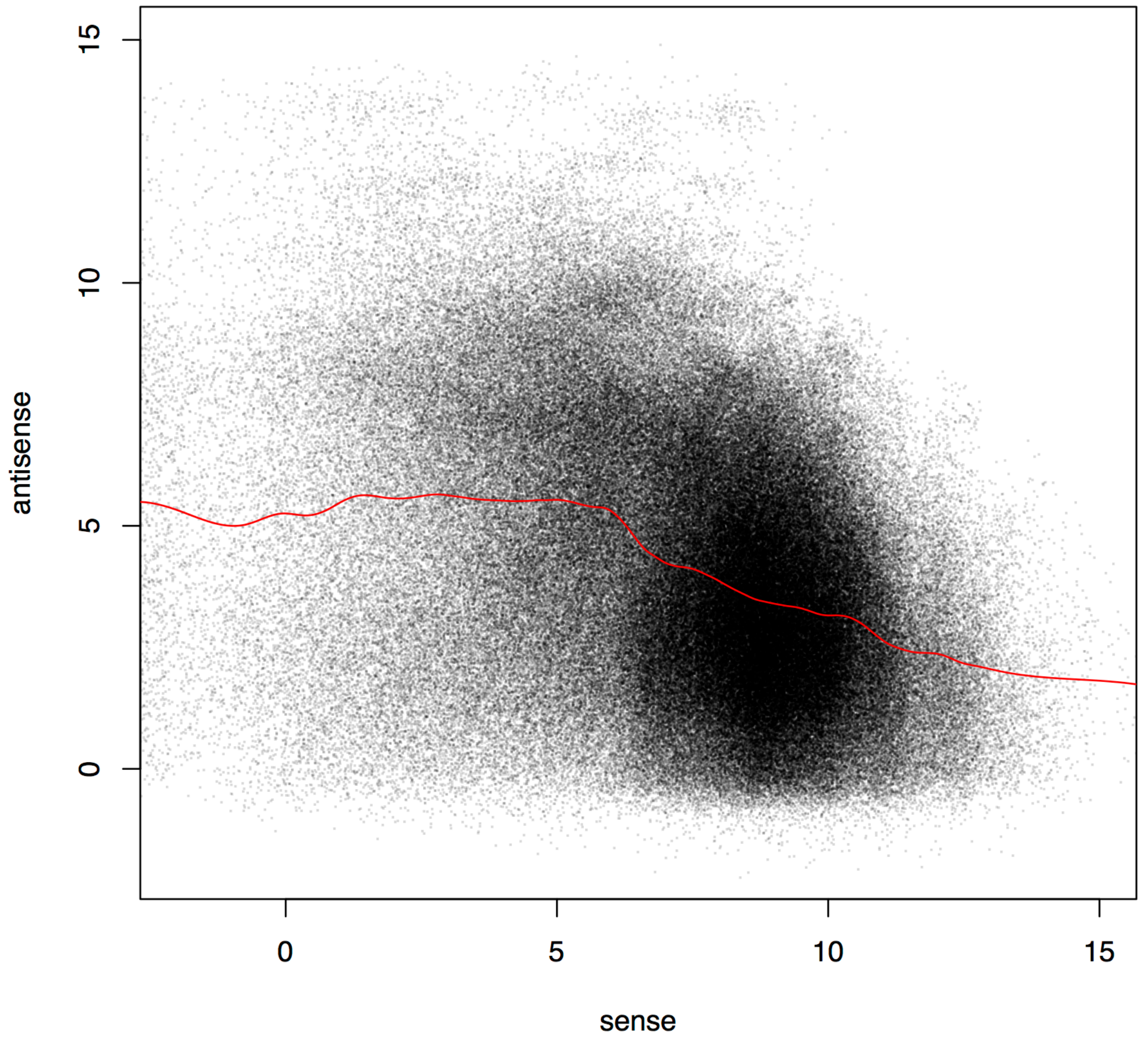

Supplementary Figure S20. Dependence of antisense gene expression levels on sense expression levels. The antisense expression level of each gene was plotted against its sense counterpart. Data of all samples were pooled. There is a significant anti-correlation between the sense and antisense levels ($\rho = -0.34$, Spearmann Rank Correaltion coefficient, $p < 2.2e^{-16}$)

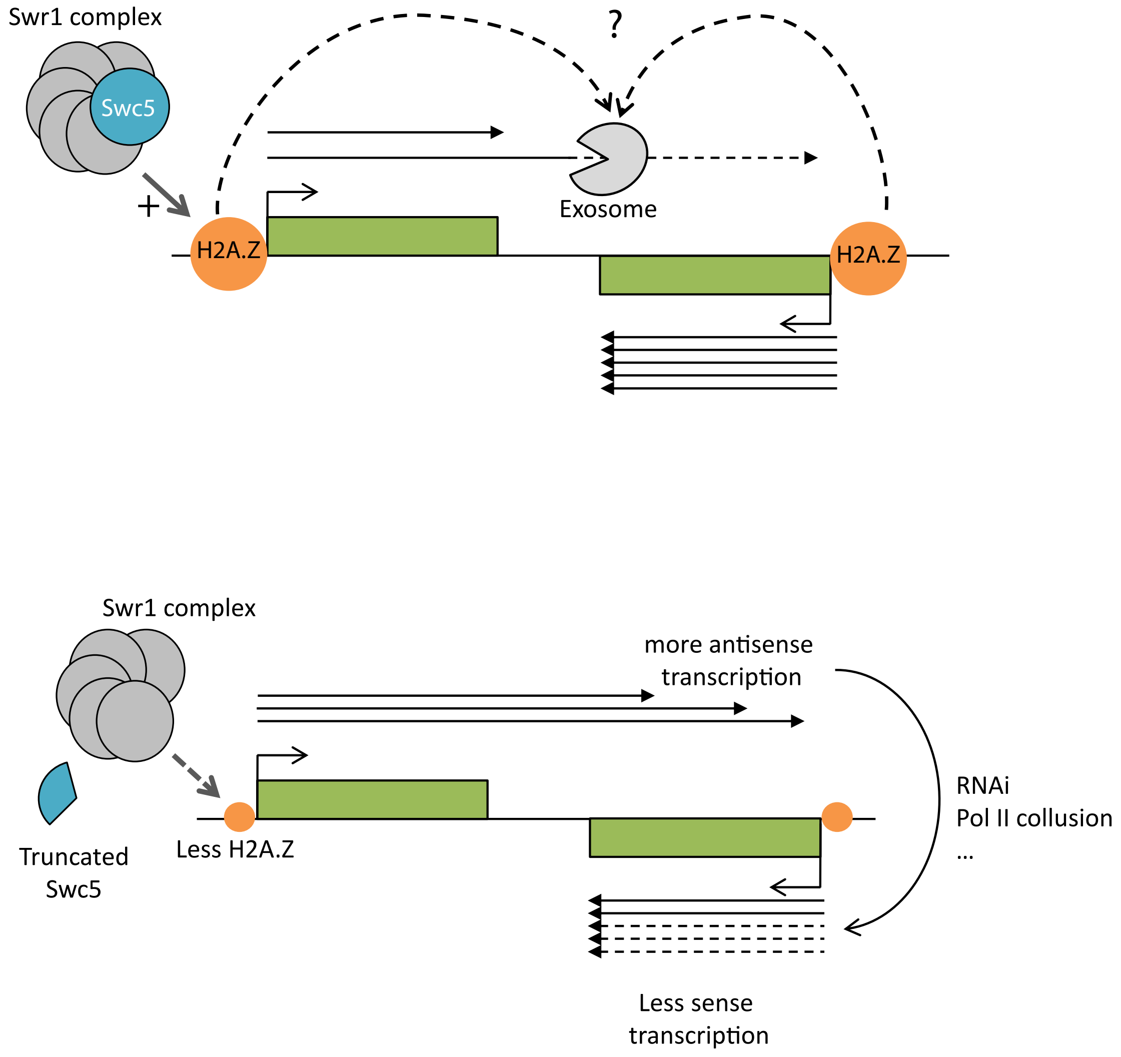

Supplementary Figure S21. Schematic representation of mechanisms for the control of read-through transcription in *swc5*[+] strains (top) and in swc5-fs strains (bottom).// The precise molecular mechanism by which H2A.Z blocks read-through presumably via the exosome remains largely unknown (Zofall et al, 2009).

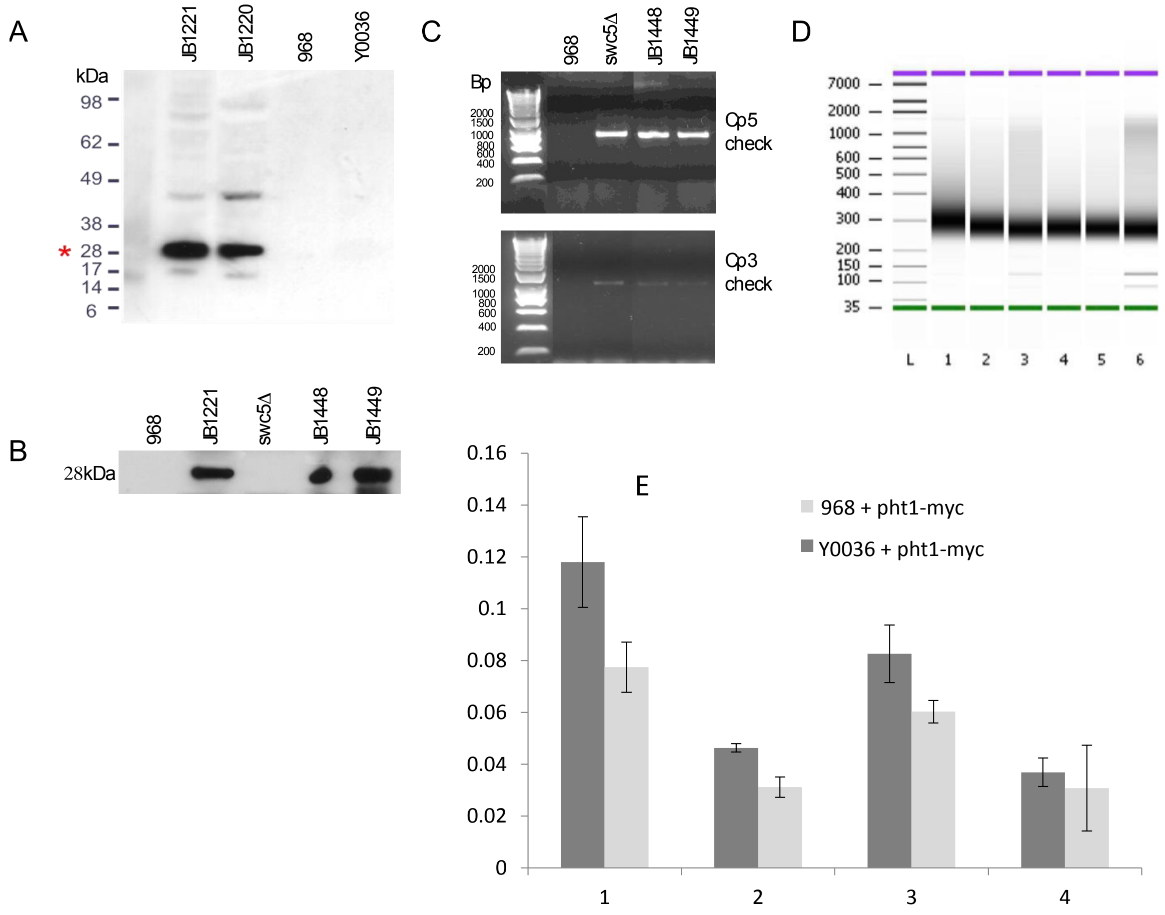

Supplementary Figure S22. ChIP-seq experiemnts quality controls

A. Western verification of C-terminal 13cmyc-tagged pht1 strains. Lanes 2 and 3 are the 13-cmyc tagged strains JB1221 and JB1220, lanes 3 and 4 equivalent untagged strains and Lane 1 -M ladder with indicated molecular weights (kDa). Pht1-13cmyc runs at ~30kDa as expected.

B. Western showing 13-cmyc tagged *Δswc5* strains JB1448 and JB1449 (lanes 5 and 6 respectively) run with appropriate controls: lane 1, 968, lane 2, JB1221 and lane 3, *Δswc5*

C. colony PCR verification of*Δswc5* strains

D. Bioanalyzer Quality Control C for 6 example ChIP-seq libaries, separated on an HS Chip (Agilent)

E. qPCR verification of ChIPs. qPCR was performed over the *adh1* locus; four regions were analysed in triplicate in 968 + *pht1-myc* compared with Y0036 + *pht1-myc* using the fast SYBR green master mix (Applied Biosystems), with serial dilutions of inputs used as standards. Data show reduced H2A.Z (Pht1) occupancy in the Y0036 strain (~100bp regions were amplified for qPCR analysis, and centered around the stated base pair with respect to the *adh1* transcription start site ATG), region 1 at -2160bp, region 2 at 0bp, region 3 at +1320bp, and region 4 at +2340bp. The means of triplicate biological repeats with standard deviations are shown. Y axis, relative quantities derived from Ct values, created using the SDS software (Applied Biosystems).

Supplementary Datasets

Supplementary Dataset S1: raw growth data
Supplementary Dataset S2: extracted growth traits
Supplementary Dataset S3: survival data
Supplementary Dataset S4: raw sense read counts from RNA-seq data
Supplementary Dataset S5: raw antisense read counts from RNA-seq data
Supplementary Dataset S6: detected genomic variation in the parental strains
Supplementary Dataset S7: genotype data for every variants for the entire library
Supplementary Dataset S8: recombination positions for every strains of the library
Supplementary Dataset S9: genotype data used for the mapping with the mapping marker position
Supplementary Dataset S10: sense eQTL mapping results (q-value of linkage for every trait and marker)
Supplementary Dataset S11: list of the significant eQTL with their genomic position, target and putative regulator
Supplementary Dataset S12: aseQTL mapping results (q-value of linkage for every trait and marker)
Supplementary Dataset S13: list of the significant aseQTL with their position and target
Supplementary Dataset S14: growth QTL results
Supplementary Dataset S15: list of eQTL that do not contain any coding gene
Supplementary Dataset S16: raw qPCR data
Supplementary Dataset S17: proteomics skylines and transition list
